# Metagenomics and metabolomics analyses of the mechanism of non-expression of natural mating behavior in captive male Malayan pangolins (*Manis javanica*)

**DOI:** 10.3389/fmicb.2026.1828282

**Published:** 2026-06-11

**Authors:** Shanghua Xu, Miaomiao Jia, Xiaobing Guo, Wenhui Liang, Yong Pan, Yuan Lin, Xinyue Li, Haitao Qiu, Defu Hu, Dingyu Yan

**Affiliations:** 1 Guangxi Key Laboratory of Special Non-Wood Forests Cultivation and Utilization, Guangxi Forestry Research Institute, Nanning, China; 2 Guangxi Forestry Laboratory, Nanning, China; 3 School of Ecology and Nature Conservation, Beijing Forestry University, Beijing, China

**Keywords:** abnormal reproduction, male Malayan pangolin, metabolome, metagenome, non-expression of natural mating behavior

## Abstract

*Ex situ* conservation and captive breeding are important measures for conserving endangered species. However, the reproduction of some wild animals, especially males, is inhibited in captivity, but the underlying mechanism has not yet been elucidated. This study aimed to investigate the microbiota and their functions, metabolites, and their metabolic pathways impacting reproduction employing metagenomics and metabolomics analyses and using male Malayan pangolins with normal (with natural mating behavior) and abnormal (no natural mating behavior) reproduction as the research objects. The results showed that the relative abundance of Proteobacteria, *Escherichia coli*, and *Shigella* spp. was significantly higher in the abnormal reproduction (AR) group. However, the relative abundance of Firmicutes and *Staphylococcus aureus* was significantly higher in the normal reproduction (NR) group. Kyoto Encyclopedia of Genes and Genomes functional pathway enrichment analysis found that citrate cycle (TCA cycle, KO00020) and pyruvate metabolism (KO00620) were significantly enriched in pangolins with AR, whereas gonadotropin-releasing hormone secretion (KO04929) was significantly enriched in pangolins with NR. Metabolites such as tryptophan, arginine, and androgen were significantly enriched in pangolins with AR, whereas L-proline, taurine, choline, and spermidine were significantly enriched in pangolins with NR. Microbiota dysbiosis, energy metabolism disorder, deficiencies in key metabolic pathways and metabolites, and hormonal disturbances are all potential factors contributing to the inability of male Malayan pangolin to express natural reproductive behavior. This study provides evidence for AR of captive pangolins and offers important insights for the conservation of captive endangered species.

## 1 Introduction

Malayan pangolins (*Manis javanica*) are widely distributed in Southeast Asia, including Myanmar, Thailand, Laos, Cambodia, Vietnam, Malaysia, Singapore, and Yunnan (China) (Cen et al., 2023; Chong et al., 2020). However, deforestation driven primarily by logging and agricultural land conversion has seriously destroyed the habitat of the Malayan pangolin (Panjang et al., 2024). This, coupled with excessive human consumption and exploitation through traditional (including folk) activities, has led to a decrease in the population of the Malayan pangolin (Chong et al., 2020; Gray et al., 2023). Pangolins are considered the most smuggled mammals across the world (Panjang et al., 2024; Yan et al., 2021). It is currently listed as a critically endangered species in the International Union for Conservation of Nature Red List of Threatened Species (Challender et al., 2019). *Ex situ* conservation and captive breeding are important measures for conserving endangered species (Mestanza-Ramón et al., 2020; Olney, 2005). However, the Malayan pangolin has low reproductive capacity, almost only giving birth to one offspring each time and only breeding once a year (Zhang et al., 2017). Moreover, it is solitary and has a large home range (Gray et al., 2023), a specialized diet, and low immunity, which are factors hindering the successful captive breeding of pangolins (Arora et al., 2023; Chin et al., 2012; Hua et al., 2015; Yusoff et al., 2016; Perera et al., 2017). This makes it one of the most difficult species to breed in captivity worldwide (Yan et al., 2021). Notably, a significant proportion of captive male Malayan pangolins fail to express natural mating behavior (Yan et al., 2021).

Research on pangolin reproduction mainly includes: reproductive behavior, gestation period and cub birth parameters, and parenting behavior of Indian pangolins (Mohapatra et al., 2015; Mohapatra et al., 2018; Mohapatra and Panda, 2014); reproductive behavior, gestation period, and cub birth parameters of Cape pangolin (Van Ee, 1966); parenting behavior and mating behavior in wild Chinese Pangolin (Pradhan and Pradhan, 2020; Sun et al., 2021); parenting behavior of wild Malayan pangolin (González-Abarzúa et al., 2025); gestation period and cub birth parameters, breeding season, and mating records of captive Chinese pangolins (Sun et al., 2024; Zhang et al., 2016). Reproductive rate, sexual maturity time, mating records, gestation period and cub birth parameters, and reproductive behavior of captive Malayan pangolins (Yan et al., 2023; Yan et al., 2021; Zhang et al., 2020); Breeding season, gestation period, and sexual maturity of captive Malayan pangolins (Zhang et al., 2015); Monitoring of reproductive hormones in male and female Chinese pangolins (Arora et al., 2023); Anatomy of the male reproductive system of Temminck’s pangolin (Tink et al., 2024); Reproductive behavior of captive Chinese pangolins (Shen et al., 2024); Study on epididymis and sperm of Chinese pangolins (Chang et al., 2020), and sperm parameters (Li et al., 2024); Sperm collection methods and sperm parameters of Malayan pangolins (Tarmizi et al., 2020).

However, few studies have mentioned the reproductive abnormalities of male pangolins, and no specific research has been conducted on this topic. Other anteaters exhibit similar characteristics, including low sociality, solitary nature, sensitivity to stress, specialized diets, and difficulty in captivity and reproduction (Knott et al., 2013). Research on the reproduction of giant anteater is extremely scarce, mainly focusing on their reproductive organs (de Moura et al., 2024), reproduction, and endocrine characteristics (Knott et al., 2013). And research on male reproductive organ characteristics and spermatogenesis (Fromme et al., 2023). Giant anteater also exhibit male non-cooperative reproductive behavior (manifested as aggression toward females) (Knott et al., 2013). Aardvarks have a low reproductive success rate in captivity, with captive populations remaining at only around 30 individuals (Fallon et al., 2026; Wojick et al., 2018). Research on aardvark reproduction includes anatomy of the male reproductive tract (Wojick et al., 2018) and monitoring of female reproductive cycle hormones and behavior (Fallon et al., 2026). Captivity does not significantly impair the reproductive system and function of male armadillos, but differences still exist compared to the wild state (Czekala et al., 1980). The limiting factors for armadillo reproduction in captivity remain unclear (Howell-Stephens et al., 2013). Current research on armadillo reproduction only includes male sperm collection and monitoring of male and female hormones (Howell-Stephens et al., 2013; Lobo et al., 2025). Echidna reproduces through a multi-male-competitive-female model (Morrow and Nicol, 2009), but under captive conditions, their reproductive behavior is difficult to meet, resulting in very poor captive breeding conditions (Wallage et al., 2015). Research on echidna reproduction includes reproductive behavior (Wallage et al., 2015), sperm collection (Morrow and Nicol, 2009), and the effects of exogenous hormone stimulation on androgens and sperm (Johnston et al., 2007).

The physiological and microbiological mechanisms underlying this behavioral inhibition remain largely unexplored. Research on pangolins focuses on their gut microbiota, primarily investigating their myrmecophagy adaptations (Ma et al., 2018; Zhang et al., 2021); differences in gut microbiota and function between Chinese and Malayan pangolins, including digestive and metabolic characteristics (Xiang et al., 2025); the impact of gut microbiota on stress, immunity, and health (Dai et al., 2024; Jiao et al., 2022); and how environmental and dietary conditions can significantly reshape the structure and function of the gut microbiota (Liu et al., 2021). None of these studies involved research on urinary microorganisms and mechanisms of abnormal reproduction.

The urogenital microbiota, the community of microorganisms inhabiting the urinary and reproductive tracts, plays a pivotal role in host physiology, while metagenomics and metabolomics, respectively, enable comprehensive characterization of microbial functional gene repertoires and of the small-molecule metabolite landscape that directly reflects physiological or pathological states (Belizário and Napolitano, 2015; Lundy et al., 2021; Wishart, 2019). Urine is particularly amenable to non-invasive collection from endangered wildlife and constitutes a valuable matrix for investigating male reproductive tract health, given that urinary microbiota dysbiosis and its associated metabolic perturbations can directly impair spermatogenesis, hormone secretion, and sexual motivation (Collino et al., 2013; Khamis et al., 2017). Building on this rationale, the present study integrated urine metagenomics and metabolomics to characterize differences in microbiota composition, functional pathways, and metabolite profiles between captive male Malayan pangolins exhibiting normal reproduction (NR) and abnormal reproduction (AR), with the goal of identifying key microbial taxa, functional pathways, and metabolites underlying the non-expression of natural mating behavior, and thereby providing mechanistic insights to inform the captive management of this critically endangered species.

## 2 Materials and methods

### 2.1 Study species, study area, and animal husbandry

Our study was conducted at the Guangxi Forestry Research Institute (22°33′N, 108°13′E), located in Nanning City, Guangxi Zhuang Autonomous Region, China. Since 2013, the institute has been engaged in the husbandry of trade-rescued pangolins. The diet consisted mainly of black ants (*Polyrhachis vicina*) and domestic silk moth (*Bombyx mori*); water was supplied ad libitum. Daily monitoring of feeding behavior enabled the early detection of poor health or illness. If pangolins did not eat for more than 2 days, a veterinarian was consulted for medical examination and treatment.

In this study, 10 captive male adult Malayan pangolins from the Guangxi Forestry Research Institute Base of Pangolin Breeding (Pangolin Base) were selected as research subjects. The animals were divided into two groups: the NR group comprising five pangolins with successful natural mating experience (able to produce offspring through natural mating after adulthood); and the AR group comprising another five male adult Malayan pangolins with no successful natural mating experience (did not produce offspring through natural mating after adulthood). Each enclosure contained eight cages, with each pangolin kept in a separate cage. All enclosures shared the same environment. The study protocol was approved by the Institutional Animal Care Committee of Guangxi Research Base of Pangolin Breeding (approval number: 2023003).

Pangolins are nocturnal animals. Therefore, they were kept in indoor cages, each consisting of three areas: an activity area (120 × 80 × 50 cm^3^), an insulated wooden winter den (40 × 35 × 28 cm^3^), and an underground summer den (40 × 35 × 28 cm^3^). A disposable plastic film was laid under the cage, and urine was collected every 2 h from 6:00 p.m. to 6:00 a.m. the next day. Supplementary Figure S1 shows photographs of the cages and a simplified diagram of the cage structure. The bottom of the cage comprised a wire mesh mat, which was suspended above the ground with slots and did not touch the ground; a plastic film was laid on the ground. The cage bottom and the plastic film were separate and did not come into direct contact. The wire mesh bottom plate was cleaned and disinfected before sample collection. If feces and urine were excreted simultaneously, the sample was considered contaminated and discarded. The plastic liner was then replaced, and urine collection was restarted until urine was successfully collected separately. A syringe was used to collect urine samples from the plastic film. The samples were sent to the laboratory on ice and stored at −80 °C until further metagenomic analysis. Metabolomics analysis was performed by centrifugation at 10,000 rpm for 10 min at 4 °C. Next, 500 μL of the supernatant was aliquoted into 1.5-mL sterile centrifuge tubes.

### 2.2 Metagenomic analysis: DNA extraction and sequencing

Further, 1,000 μL of CTAB lysis buffer was pipetted into a 2.0-mL EP tube, mixed with lysozyme and an appropriate amount of the sample, and incubated in a 65 °C water bath. The mixture was gently inverted several times during incubation to ensure complete lysis of the sample. Next, the sample was centrifuged to obtain the supernatant, which was mixed with phenol (Ph8.0):chloroform:isoamyl alcohol (25:24:1) by inversion and centrifuged at 12,000 rpm for 10 min. The supernatant was pipetted into a 1.5-mL centrifuge tube, mixed with isopropanol, shaken up and down, and precipitated at −20 °C. It was centrifuged again at 12,000 rpm for 10 min. The supernatant was carefully discarded without disturbing the precipitate. The pellet was washed twice with 1 mL of 75% ethanol. Residual liquid was collected by centrifugation and then sucked out with a pipette. The pellet was blown dry on a clean bench or air-dried at room temperature, taking care to avoid over-drying, as excessively dried DNA is difficult to dissolve. Then, ddH_2_O was added to dissolve the DNA sample. The sample was incubated at 55 °C–60 °C for 10 min to aid dissolution. Further, 1 μL of RNase A was added to digest RNA, followed by incubation at 37 °C for 15 min.

DNA concentration was measured using a Qubit dsDNA Assay Kit in a Qubit 2.0 Flurometer (Life Technologies, CA, United States). The optical density value was 1.8–2.0. DNA contents above 1 μg were used to construct the library.

A total of 1 μg of DNA per sample was used as the input material for library preparation. Sequencing libraries were generated using the NEBNext Ultra DNA Library Prep Kit for Illumina NovaSeq 6000 (Illumina, San Diego, CA, United States) following the manufacturer’s protocols. The index codes were added to attribute sequences for each sample. Briefly, the DNA sample was fragmented by sonication to an average size of 350 bp, and then the DNA fragments were end-repaired, A-tailed, and ligated with the full-length Illumina sequencing adapters, followed by polymerase chain reaction (PCR) amplification. Finally, PCR products were purified [AMPure XP system (Beckman Coulter Life Sciences, CA, United States)]. The libraries were then analyzed for size distribution using Agilent 2100 Bioanalyzer (Agilent Technologies, Santa Clara, CA, United States) and quantified using real-time PCR.

The clustering of the index-coded samples was performed on a cBot cluster generation system (Illumina, San Diego, CA, United States) following the manufacturer’s protocols. After cluster generation, the libraries were sequenced on an Illumina NovaSeq 6000 (Illumina, San Diego, CA, United States) and paired-end reads were generated.

### 2.3 Metabolomics: extraction, HPLC conditions, and MS conditions

The sample stored in a refrigerator at −80 °C was thawed on ice and vortexed for 10 s. A 200-μL extract solution (ACN:methanol = 1:4, *v*/*v*) containing internal standard was added to 200 μL of the sample (Supplementary Table S1). Then, the sample was vortexed for 3 min and centrifuged at 12,000 rpm for 10 min (4 °C). A total of 350 μL of the supernatant was collected and completely concentrated. A 100-μL solution (methanol:water = 7:3, *v*/*v*) was used to reconstitute the residual, vortexed for 3 min, and then sonicated for 10 min in an ice water bath. The sample was then centrifuged at 12,000 rpm for 3 min (4 °C). Next, 80-μL aliquots of the supernatant were transferred for LC–MS analysis.

All samples were acquired using the LC–MS system (SCIEX, Framingham, MA, United States), following the manufacturer’s protocols. The analytical conditions were as follows: UPLC: Waters ACQUITY UPLC BEH C18 column, 1.8 μm, 2.1 mm × 100 mm; column temperature, 40 °C; flow rate, 0.4 mL/min; injection volume, 2 μL; and solvent system, water (0.1% formic acid):acetonitrile (0.1% formic acid). The column was eluted with 5% mobile phase B (0.1% formic acid in acetonitrile) in 0 min, followed by a linear gradient to 90% mobile phase B (0.1% formic acid in acetonitrile) for 11 min, held for 1 min, returned to 5% mobile phase B in 0.1 min, held for 1.9 min, and then rapidly returned to starting conditions.

Data acquisition was performed in information-dependent acquisition mode using Analyst TF 1.7.1 Software (Sciex, ON, Canada). The source parameters were set as follows: ion source gas 1 (GAS1), 50 psi; ion source gas 2 (GAS2), 50 psi; curtain gas (CUR), 35 psi; temperature (TEM), 550 °C (positive mode) or 450 °C (negative mode); declustering potential (DP), 60 V (positive mode) or −60 V (negative mode); and ion spray voltage floating, 5,000 V (positive mode) or −4,000 V (negative mode). The TOF-MS scan parameters were as follows: mass range, 50–1,000 Da; accumulation time, 200 ms; and dynamic background subtraction, enabled. The product ion scan parameters were as follows: mass range, 25–1,000 Da; accumulation time, 40 ms; collision energy, 30 V (positive mode) or −30 V (negative mode); collision energy spread, 15; resolution, UNIT; charge state, 1 to 1; intensity, 100 cps; isotopes excluded within 4 Da; mass tolerance, 50 mDa; and maximum number of candidate ions to monitor per cycle, 12.

### 2.4 Bioinformatics and statistical analysis

#### 2.4.1 Metagenomics

##### 2.4.1.1 Pre-processing of sequencing results

Fastp software was used for raw data quality control, default software parameters were selected to preprocess raw data obtained using the Illumina NovaSeq 6000 (Illumina, San Diego, CA, United States) sequencing platform, and clean data were obtained for subsequent analysis. If contamination was found in the sample, it was compared with the host database to filter out reads from the host (Karlsson et al., 2012; Scher et al., 2013). Bowtie2 was used for sequence alignment, with parameters set to --sensitive, -I 200, -x 400.

##### 2.4.1.2 Metagenome assembly

After pretreatment, clean data were obtained, and assembly analysis was performed using MEGAHIT Assembly software. Assembly parameters were as follows: -- Kmin 35 -- K-max 95 -- K-Step 20 -- min-contig-Len 500. The clean data of each sample after quality control were compared to contigs of each sample after assembly using the Bowtie2 software to obtain PE reads that were not utilized. Comparison parameters were as follows: -I 200, -x 400. Unutilized reads of each sample were put together for mixed assembly (Karlsson et al., 2012; Qin et al., 2010). The assembly parameters were the same as those of a single sample.

##### 2.4.1.3 Gene prediction and abundance analysis

Based on contigs (≥500 bp) of each sample and mixed assembly, MetaGeneMark was used to predict open reading frame (ORF) (Oh et al., 2014; Qin et al., 2014), employing the default parameters.

Based on the predicted results, the predicted genes with a length of less than 100 nucleotides were filtered out. The ORF prediction results of all samples and mixed assembly were combined, and CD-HIT software was used to remove redundancy, so as to obtain the nonredundant initial gene catalog (here, operationally, the nucleic acid sequence encoded by nonredundant continuous genes is called genes). By default, a sequence identity threshold of 95% and a coverage threshold of 90% were used for clustering, and the longest sequence in each cluster was selected as the representative sequence. The parameters were set as follows: -c 0.95, -g 0, -AS 0.9, -g 1, -d 0;

Bowtie2 was used to compare the clean data of each sample with the initial gene catalog, and the number of reads in each sample was calculated. --end-to-end, --sensitive, -I 200, -x 400. Genes supporting reads ≤22 in each sample were filtered out to obtain a gene catalog (unigenes) for subsequent analysis.

Gene abundance in each sample was calculated based on the number of mapped reads and the corresponding gene length, as shown in the following formula:

$G_{k}$ = $\frac{r_{k}}{L_{k}}\cdot \frac{1}{\sum_{i=1\;}^{n}\frac{r_{i}}{L_{i}}}$

where *r* is the number of reads compared to the gene, and *L* is the length of the gene.

##### 2.4.1.4 Species annotation

Comparison between genes and species database: DIAMOND software was used to compare unigene sequences with those of bacteria, fungi, archaea, and viruses extracted from the National Center for Biotechnology Information (NCBI) NR database (BLastp, EVALue ≤ 1E-5) (Buchfink et al., 2015).

LCA algorithm: Each sequence may have multiple comparison results, and therefore multiple data points on species classification were obtained. The LCA algorithm of the MEGAN software was used to obtain the information on the final species annotation of the sequence (Huson et al., 2011).

Based on the results of LCA annotation and gene abundance, the abundance of each sample at different taxonomic levels (phylum and genus) was determined. The abundance of a given taxon in a sample was calculated as the sum of the abundances of all genes annotated to that taxon.

##### 2.4.1.5 Annotation using common functional databases

Sequence alignment: Unigenes were compared with each functional database using DIAMOND software (BLastP, EVALue ≤ 1E-5).

Filtering of comparison results: For each sequence, DIAMOND was used with the parameter—max-target-seqs 1 to retain only the top alignment result. Based on the annotation results, the relative abundance of different functional levels was calculated (the relative abundance of each functional level was equal to the sum of the relative abundance of all genes assigned to that functional category). The KEGG database was hierarchically classified into six levels for functional analysis.

Based on the results of functional annotation and gene abundance, a gene count table for each sample at each functional classification level was generated. The number of genes with a certain function in a sample was defined as the number of genes annotated to that function with a non-zero abundance.

Based on the abundance at each classification level, the following analyses were performed: statistics of annotated gene counts, overview of relative abundance, abundance-based clustering heat maps, principal component analysis (PCA) and NMDS for dimensionality reduction, ANOSIM to assess inter-group differences based on functional abundance, and comparative analysis of metabolic pathways. This was followed by Metastat analysis of functional differences between groups. Both Shannon and Simpson diversity indexes were calculated using QIIME 2 (version 1.8.0). This analysis used Simpson’s Diversity Index (1−D) (The Wilcoxon rank-sum test was used to assess differences between the two groups). Adonis is a nonparametric permutational multivariate analysis of variance method based on the Bray–Curtis distance. Adonis analyses were performed using the Adonis function in the vegan R package.

#### 2.4.2 Metabolomics

The original data file acquired using LC–MS was converted into mzXML format with ProteoWizard software. Peak extraction, peak alignment, and retention time correction were performed using the XCMS program. The SVR method was used to correct the peak area. The peaks with a detection rate lower than 50% in each group of samples were discarded. Then, information on metabolic identification was obtained by searching the laboratory’s self-built database, integrated public database, AI database, and metDNA.

##### 2.4.2.1 PCA

Unsupervised PCA was performed using the statistics function prcomp within R.^1^ The data were unit variance scaled before unsupervised PCA.

##### 2.4.2.2 Hierarchical cluster analysis and Pearson correlation coefficients

The hierarchical cluster analysis (HCA) results of samples and metabolites were presented as heatmaps with dendrograms. Pearson correlation coefficients (PCCs) between samples were calculated using the cor function in R and presented as only heatmaps. Both HCA and PCC were performed using the R package ComplexHeatmap. For HCA, the normalized signal intensities of metabolites (unit variance scaling) were visualized as a color spectrum.

##### 2.4.2.3 Selection of differential metabolites

For two-group analysis, differential metabolites were determined using VIP (VIP > 1) and *p* value (*p* value < 0.05, Student *t* test). VIp values were extracted from the OPLS-DA result, which also contained score plots and permutation plots generated using the R package MetaboAnalystR. The data were log-transformed (log2) and mean-centered before OPLS-DA. A permutation test (200 permutations) was performed to avoid overfitting.

##### 2.4.2.4 KEGG annotation and enrichment analysis

Identified metabolites were annotated using the KEGG Compound database.^2^ The annotated metabolites were then mapped to the KEGG Pathway database^3^ (Kanehisa et al., 2017). Significantly enriched pathways were identified with a hypergeometric test’s *P* value for a given list of metabolites.

## 3 Results

After quality control and host gene removal, 1,584,583,258 reads were retained for metagenomic sequencing, with 158,458,325 ± 21,973,057 (mean ± standard error of the mean) reads per sample. After reassembly, 1,266,462 contigs (N50 length of 1,535 ± 50 bp) were generated, with an N50 length of 20,358 ± 2,895 per sample. The urine metagenome comprised 95.94% bacteria, 3.43% viruses, 0.36% eukaryotes, and 0.08% archaea. Metastats analysis was used to compare the microbiota communities between the two groups, revealing significant differences in bacteria, archaea, and eukaryotes (*p* < 0.05, Table 1), whereas viral communities did not differ significantly between groups (*p* > 0.05).

**Table 1:** Microbiota composition of the urine of male Malayan pangolins.

Taxonomy	NR1	NR2	NR3	NR4	NR5	AR1	AR2	AR3	AR4	AR5	*P* value
Others	0.25093	0.30785	0.21848	0.21421	0.20691	0.14303	0.14367	0.14261	0.14335	0.14469	0.0038
Archaea	0.00024	0.00030	0.00022	0.00023	0.00020	0.00019	0.00019	0.00020	0.00020	0.00020	0.0133
Bacteria	0.73187	0.66776	0.76684	0.77096	0.77888	0.83831	0.83774	0.83872	0.83808	0.83683	0.005
Eukaryotes	0.00273	0.00253	0.00173	0.00166	0.00161	0.00062	0.00064	0.00063	0.00064	0.00063	<0.001
Viruses	0.01424	0.02155	0.01272	0.01294	0.01240	0.01785	0.01776	0.01783	0.01773	0.01766	0.0997

### 3.1 The pangolins in the AR group exhibited higher microbial diversity and a higher abundance of pathogenic bacteria in their urine

**Figure 1:**
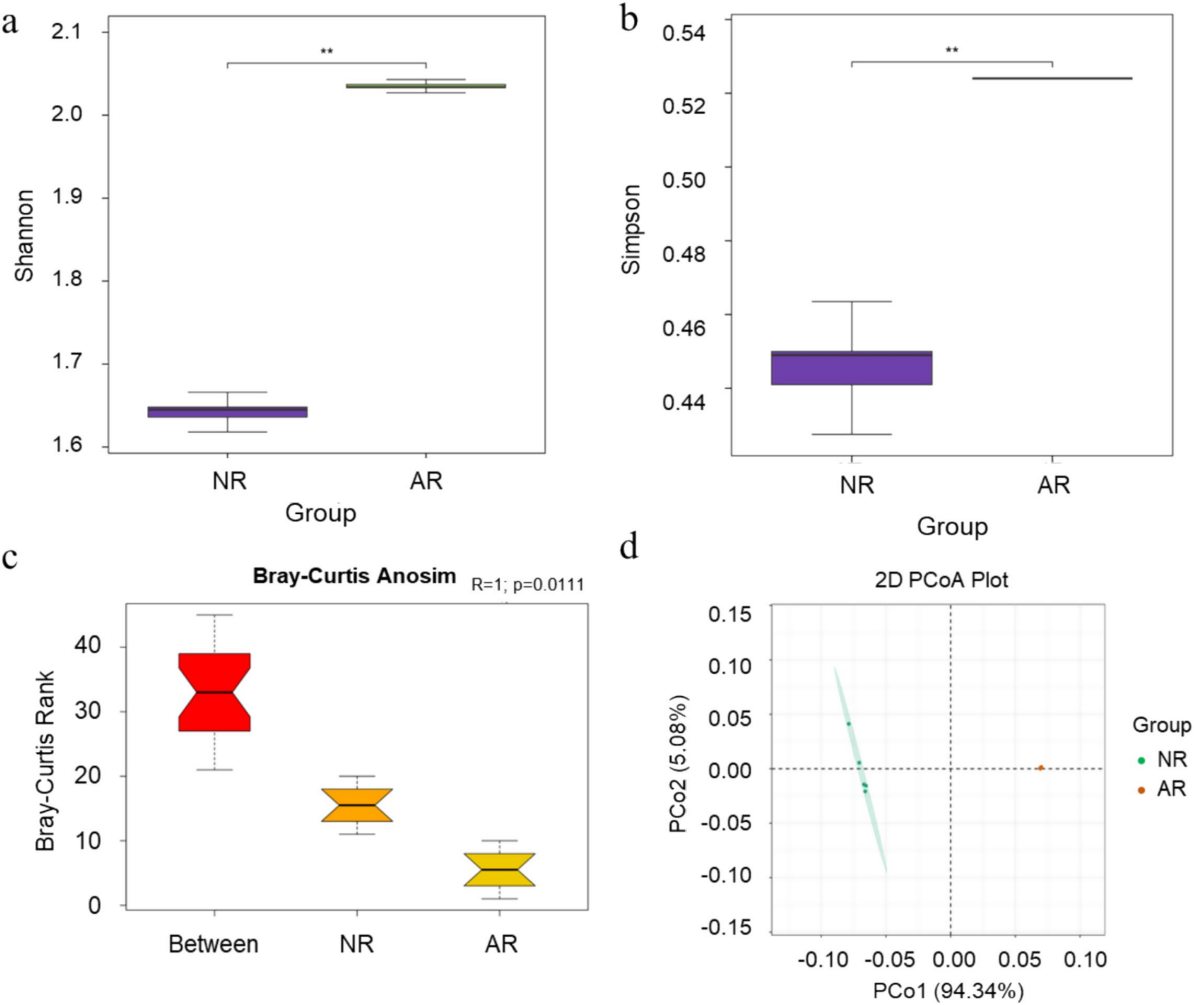
Differences in the taxonomic composition of urine microbiota between pangolins with NR and pangolins with AR. **(a,b)** Differences in α diversity (Shannon and Simpson indices). Box plot of differences in α diversity based on species abundance between groups. **(c)** Based on the ANOSIM analysis of the Bray–Curtis distance of species abundance, the *R* value was between (−1, 1). An *R* value greater than 0 implied a significant difference between groups. An *R* value less than 0 implied that the difference within the group was greater than the difference between the groups. The credibility of the statistical analysis was expressed by the *p* value, and *p* < 0.05 indicated that the statistics were significant. **(d)** PCoA results of β diversity based on species level.

We investigated the differences in the structural diversity of the urine microbiota between pangolins with NR and AR using Shannon and Simpson indices to assess the microbial α diversity, respectively. We found that the α diversity of pangolins with AR was significantly higher than that of pangolins with NR (Shannon index, Wilcoxon rank-sum test, *p* < 0.01, Figure 1a; Simpson index between the two groups, Wilcoxon rank-sum test, *p* < 0.01, Figure 1b). The results showed that the urine microbial diversity of pangolins with AR was significantly higher than that of pangolins with NR.

The results of ANOSIM analysis based on the Bray–Curtis distance of species abundance showed that the difference between pangolins with NR and pangolins with AR was greater than the difference within the group (ANOSIM test, *R* = 1, *p* = 0.0111, Figure 1c), and the grouping was meaningful. The results of principal coordinate analysis (PCoA) analysis based on the Bray–Curtis distance of species abundance showed that the microbiota between pangolins with NR and pangolins with AR could be clearly clustered (Figure 1d). Also, the Adonis analysis showed a significant difference in the composition of the microbiota between pangolins with NR and pangolins with AR (Adonis analysis, *R*^2^ = 0.059, *p* < 0.01), indicating that the β diversity of the microbiota in pangolins with NR was different from that in pangolins with AR.

**Figure 2:**
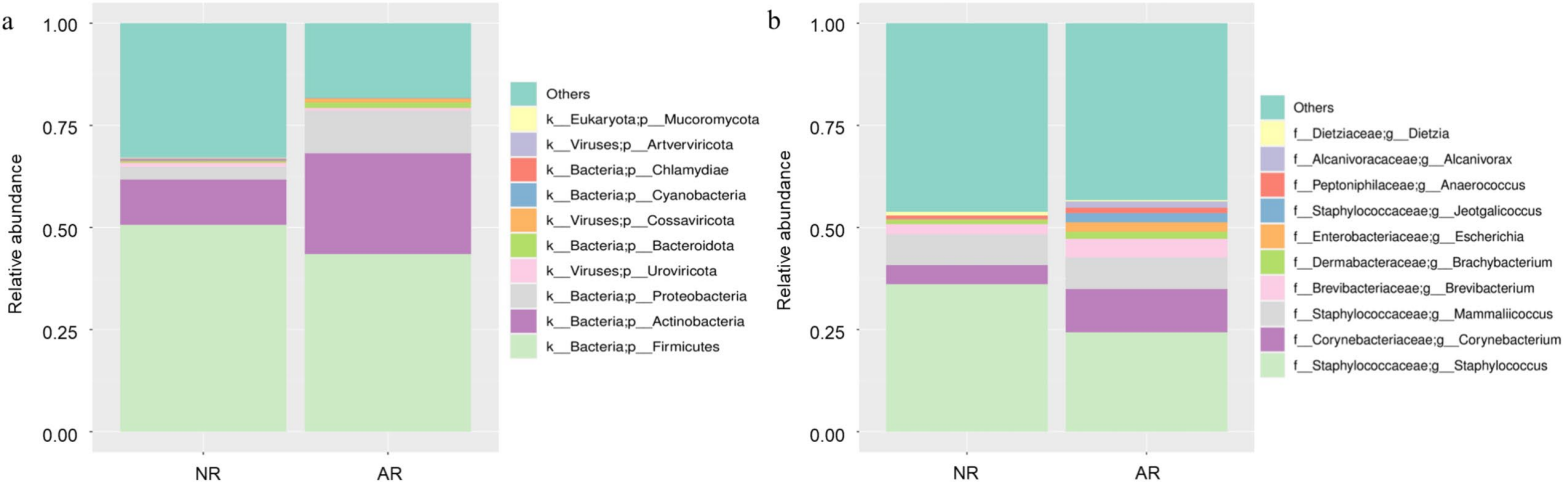
Bar graphs depicting species relative abundance at the phylum and genus levels (group). **(a)** Relative abundance bar graph at the phylum level. **(b)** Relative abundance bar graph at the genus level.

The dominant microbial phyla included Firmicutes (47.06% ± 5.90%), followed by Actinobacteria (17.89% ± 7.18%) and Proteobacteria (6.76% ± 3.90%). The dominant genus included *Staphylococcus* spp. (30.21% ± 7.24%), followed by *Corynebacterium* spp. (7.65% ± 3.12%), *Mammaliicoccus* spp. (7.62% ± 0.57%), and *Brevibacterium* spp. (3.56% ± 1.09%). The dominant bacterial species were *Mammaliicoccus lentus* (5.14% ± 0.52%), *Staphylococcus cohnii* (1.47% ± 0.34%), *Staphylococcus simulans* (1.38% ± 0.25%), *Mammaliicoccus sciuri* (1.21% ± 0.29%), and *Corynebacterium* var*iabile* (1.18% ± 0.87%) (Figures 2a,b).

**Figure 3:**
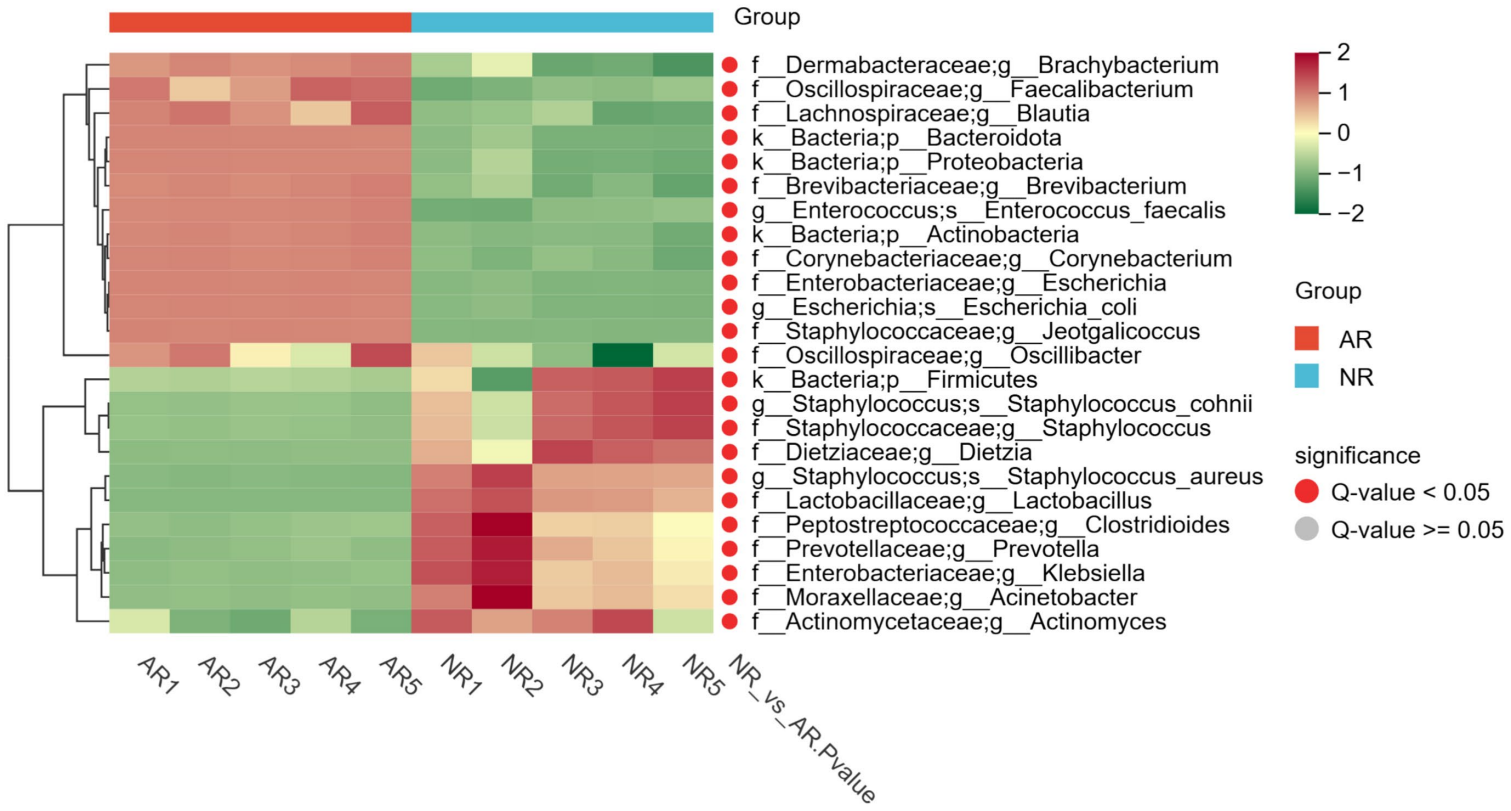
Differences in the relative abundance of microbial phyla, genera, and species in the urinary microbiota of pangolins with NR and those with AR.

Metastats analysis further confirmed the differences in microbiota between the two groups. The comparative analysis of differential abundance at the phylum level revealed that the abundance of Firmicutes was significantly higher in the urine of pangolins with NR (*p* < 0.05), whereas the abundance of Actinobacteria (*p* < 0.05), Proteobacteria (*p* < 0.05), and Bacteroidota (*p* < 0.05) was significantly higher in the urine of pangolins with AR (Metastats analysis, *p* < 0.05; Figure 3). These phylum-level differences demonstrate a significant shift toward potentially pathogenic and proinflammatory phyla in AR pangolins, with Firmicutes preferentially enriched in NR individuals.

At the genus level, the abundance of *Staphylococcus* spp., *Dietzia* spp., *Klebsiella* spp., *Clostridioides* spp., *Acinetobacter* spp., *Lactobacillus* spp., *Prevotella* spp., and *Actinomyces* spp. was significantly higher in the NR group than in the AR group. However, the abundance of *Corynebacterium* spp., *Brevibacterium* spp., *Brachybacterium* spp., *Escherichia* spp., *Jeotgalicoccus* spp., *Oscillibacter* spp., *Blautia* spp., and *Faecalibacterium* spp. was significantly higher in the AR group (Metastats analysis, *p* < 0.05; Figure 3). At the genus level, NR pangolins were enriched with commensal and potentially protective genera, while AR pangolins showed predominance of opportunistic pathogens and dysbiosis-associated taxa, suggesting that the microbial balance in their urogenital tracts may have been disrupted. These species-level differences highlight a greater urogenital infection risk in AR pangolins, evidenced by the significant elevation of pathogenic *Escherichia coli* and *Enterococcus faecalis* compared to the NR group.

At the species level, the abundance of *S. cohnii* and *Staphylococcus aureus* (*p* < 0.05) was significantly higher in the NR group, whereas the abundance of *Escherichia coli* and *Enterococcus faecalis* (*p* < 0.05) was significantly higher in the AR group (Metastats analysis, *p* < 0.05; Figure 3).

### 3.2 Energy metabolism pathways are predominantly enriched in AR pangolins, while GnRH secretion and neurosynaptic pathways are preferentially active in NR pangolins

**Figure 4:**
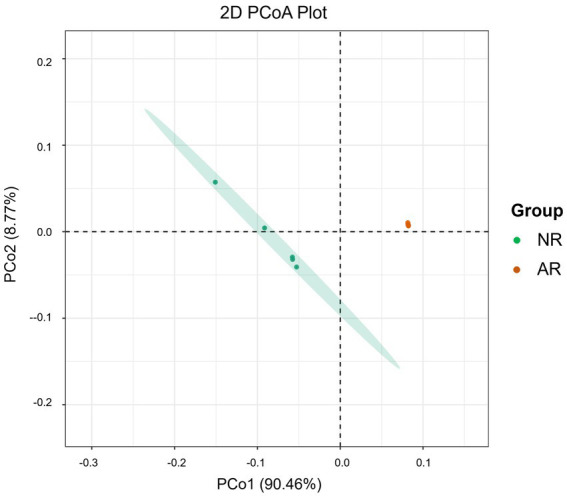
Differences in the functional gene composition of urine microbiota. PCoA of pangolins with NR and pangolins with AR based on relative gene abundance in urine microbiota (Bray–Curtis distance; Adonis analysis, *R*^2^ = 0.171, *p* < 0.01). The ellipse borders represent the 95% confidence interval.

The function of the urine microbiota was confirmed using metagenomic sequencing. Based on the Kyoto Encyclopedia of Genes and Genomes (KEGG) database, 8,038 KEGG orthologous (KO) genes were identified. PCoA based on the Bray–Curtis distance of the relative abundance of KO genes revealed separation between pangolins with NR and pangolins with AR (Adonis analysis, *R*^2^ = 0.171, *p* < 0.01; Figure 4). Moreover, Metastats analysis found that the enrichment of 345 KEGG Level 3 pathways was significantly different between pangolins with NR and pangolins with AR.

**Figure 5:**
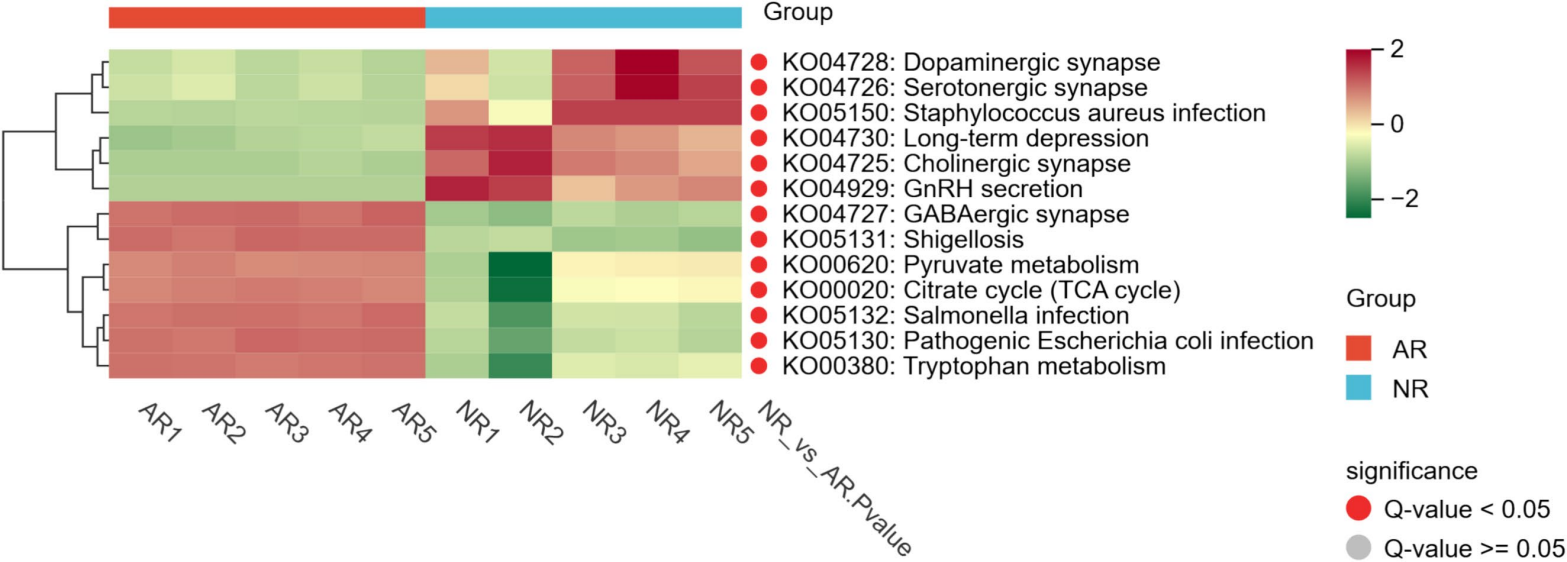
Differences in KEGG level 3 pathway enrichment of pangolins with NR and AR.

KEGG Level 3 pathways, such as dopaminergic synapse (KO04728), cholinergic synapse (KO04725), serotonergic synapse (KO04726), gonadotropin-releasing hormone (GnRH) secretion (KO04929), long-term depression (KO04730), and *S. aureus* infection (KO05150), were significantly more enriched in pangolins with NR. However, the citric acid cycle (TCA cycle) (KO00020), pyruvate metabolism (KO00620), GABAergic (γ-aminobutyric acid) synapse (KO04727), pathogenic *E. coli* infection (KO05130), *Shigella* infection (KO05131), *Salmonella* infection (KO05132), and tryptophan metabolism (KO00380) were significantly enriched in pangolins with AR (Metastats analysis, *p* < 0.05; Figure 5).

**Figure 6:**
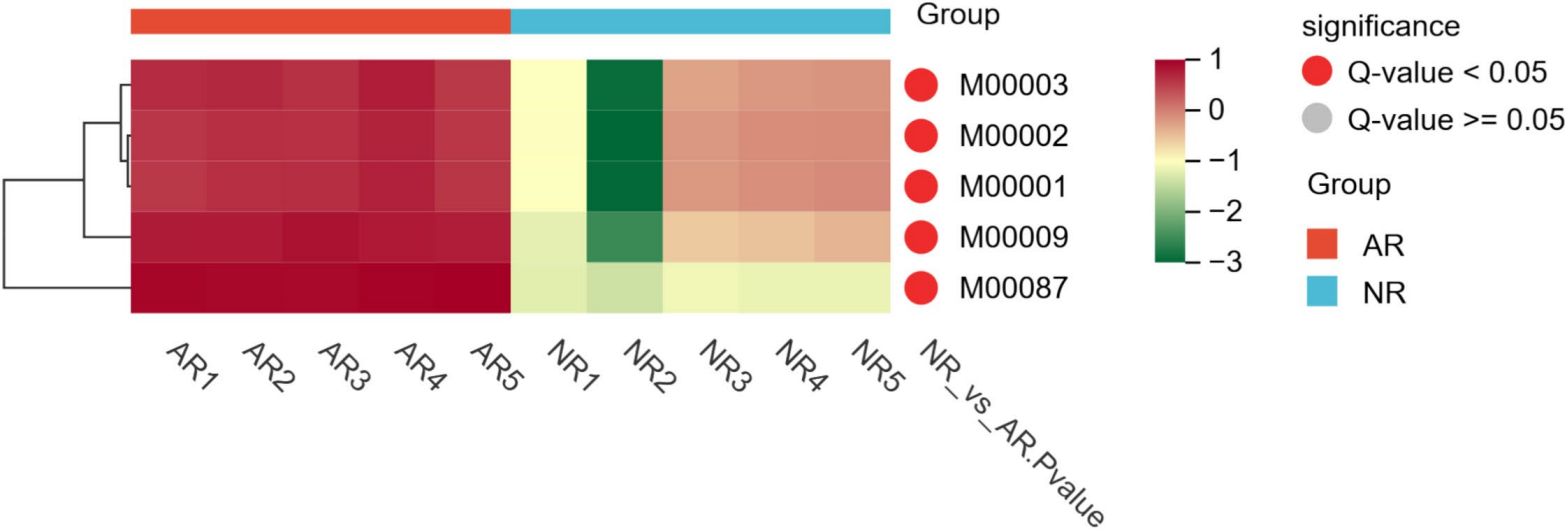
Differences in module enrichment of pangolins with NR and AR.

Further analysis of the metagenomic KO genes revealed that the abundance of 5,476 KO genes was significantly different between pangolins with NR and pangolins with AR. The KEGG Mapper Color tool was used to compare these 5,476 KO genes. After screening these KO genes by metabolic modules, it was found that M00001 Glycolysis (Embden–Meyerhof pathway; glucose ≥ pyruvate), M00002 Glycolysis (core module involving three-carbon compounds), M00003 Gluconeogenesis (oxaloacetate ≥ fructose-6P), M00009 Citrate cycle (TCA cycle, Krebs cycle), M00087 beta-Oxidation were significantly enriched in pangolins with AR (Figure 6). The significant enrichment of core glycolytic, gluconeogenic, and oxidative metabolic modules in AR pangolins is consistent with compensatory upregulation of energy metabolism in response to chronic energy insufficiency.

### 3.3 NR pangolins show enrichment of reproductive-beneficial metabolites, while AR pangolins display disturbed hormonal and metabolic profiles

**Figure 7:**
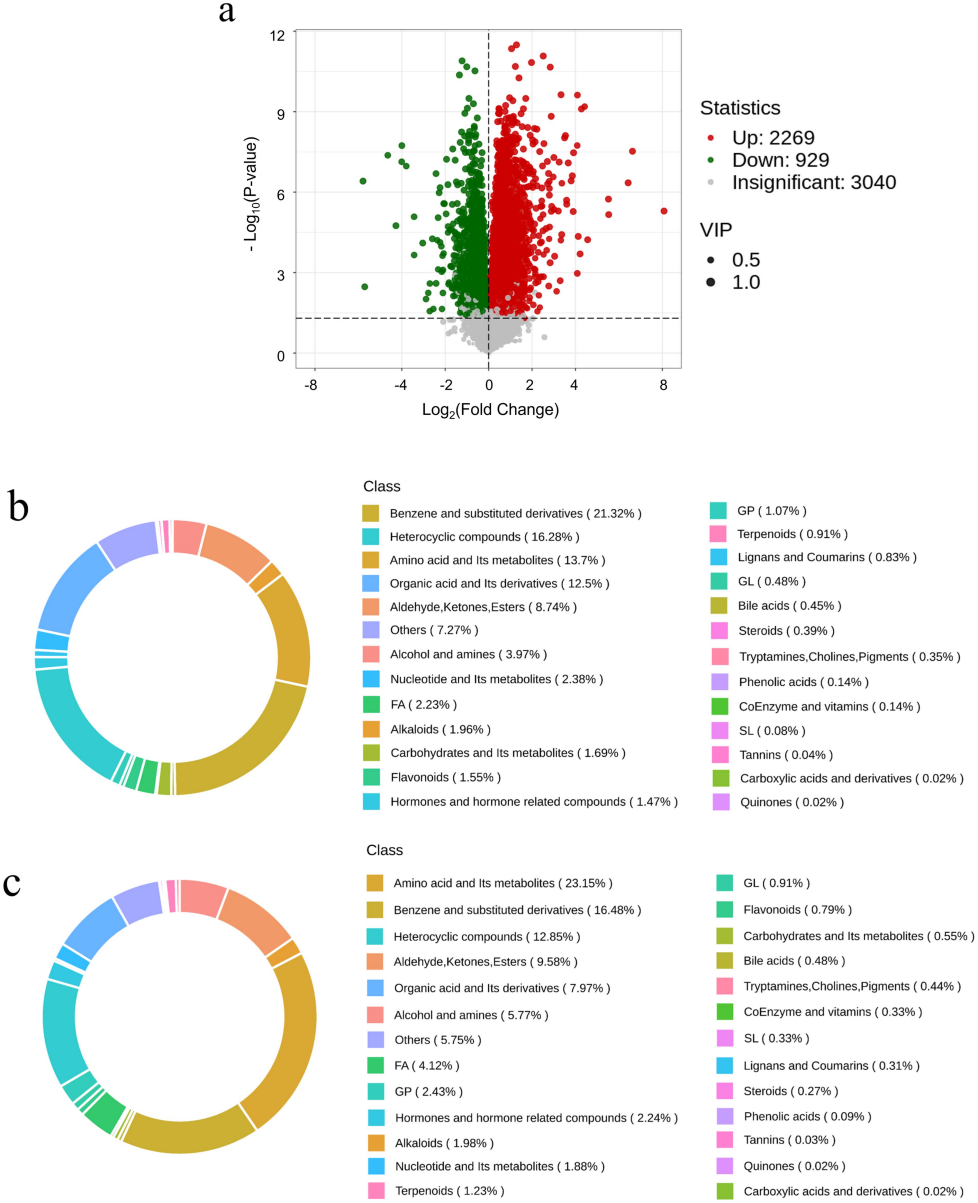
Metabolite profiles. **(a)** VIp value graph of differential metabolites. **(b)** Ring diagram of metabolite class composition in negative ion mode. **(c)** Ring diagram of metabolite class composition in positive ion mode.

We employed LC–MS/MS to analyze metabolites in all pangolin urine samples using a nontargeted metabolomics approach. The peaks detected in the samples were read, and the peak area of each characteristic ion was used as the relative quantitative measure of the corresponding metabolite. The quantitative results were normalized using the total peak area. A total of 9,596 different metabolites were obtained, of which 5,345 were obtained in positive ion mode and 4,251 in negative ion mode (Table 2; Figure 7a). Among these, 3,198 were significantly different between the 2 groups.

**Table 2:** Summary of metabolite identification.

	All	C18 positive	C18 negative
Metabolite count	9596.00	5345.00	4251.00

	Total	Down	Up
Significant metabolite count	3198.00	929.00	2269.00

After comparing the metabolites with the Human Metabolome Database (HMDB), 4,292 metabolites belonging to 95 subclasses were annotated (Figures 7b,c). These metabolites were mainly benzene and its substituted derivatives (18.9%), amino acids and their metabolites (18.43%), heterocyclic compounds (14.57%), organic acids and their derivatives (10.24%), aldehydes, ketones, and esters (9.16%).

**Figure 8:**
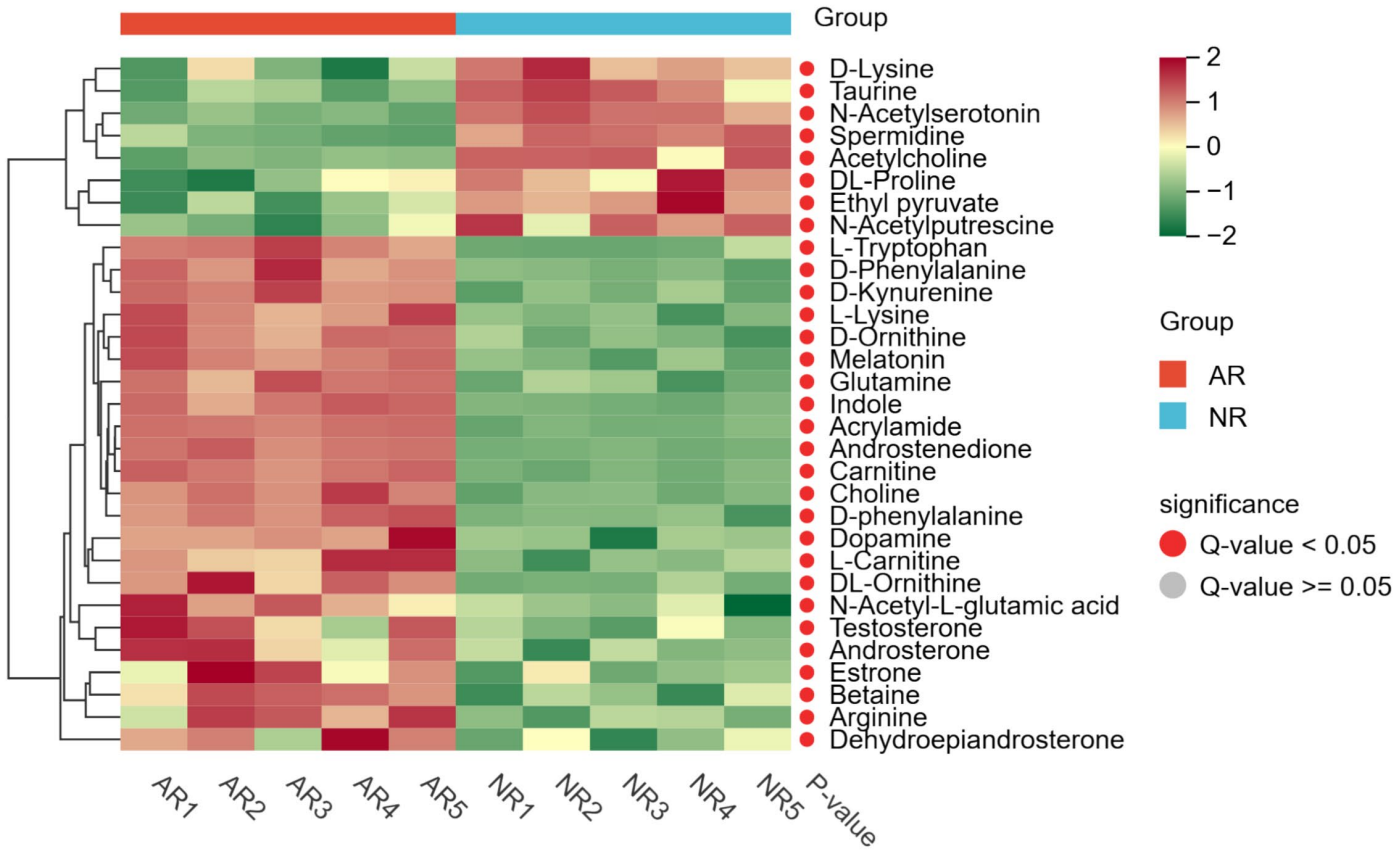
Differences in metabolites of pangolins with NR and AR.

Metabolites such as tryptophan, kynurenine, arginine, cholic acid, L-carnitine, dopamine, indole, acrylamide, androsterone, testosterone, dehydroepiandrosterone, estrone, and androstenedione were significantly enriched in pangolins with AR (*p* < 0.05). However, acetylcholine, *N*-acetylserotonin (NAS), spermidine, *N*-acetylputrescine, taurine, L-proline, pyruvate, spermidine, taurine, choline, and betaine were significantly enriched in pangolins with NR (*p* < 0.05) (Figure 8). These data reveal a clear metabolic divergence between NR and AR pangolins, with AR pangolins showing elevated stress- and catabolism-associated metabolites, while NR pangolins are enriched with neuroactive and reproductive-supportive compounds.

These metabolites were compared with the KEGG database, and 1,124 metabolites were found to be significantly different between the NR and AR groups (FC > 1.5 or FC < 0.7, VIP > 1, *p* < 0.05). Among the 1,124 metabolites with significant differences in abundance, the abundance of 362 metabolites was significantly higher in the NR group than in the AR group, whereas the abundance of 762 metabolites was significantly higher in the AR group than in the NR group. The KEGG Mapper Color tool was used to compare these 1,124 differential metabolites with the differentially expressed microbial genes identified by metagenomic analysis, resulting in the identification of 301 metabolic pathways.

We used the KEGG Mapper Color tool to combine the aforementioned 1,124 differential metabolites with the differential genes screened by metagenomic analysis. We found that AR were significantly enriched in M00133 Polyamine biosynthesis, arginine ≥ agmatine ≥ putrescine ≥ spermidine, whereas M00037 Melatonin biosynthesis, tryptophan ≥ serotonin ≥ melatonin, M00038 Tryptophan metabolism, and tryptophan ≥ kynurenine ≥ 2-aminomuconate were significantly enriched in pangolins with NR. The metabolic pathways, including M00134 Polyamine biosynthesis, arginine ≥ ornithine ≥ putrescine, M00977 C19-Steroid hormone biosynthesis (androgen backdoor pathway), pregnenolone ≥ androsterone ≥ dihydrotestosterone, M00976 C19-Steroid hormone biosynthesis, pregnenolone ≥ testosterone ≥ dihydrotestosterone, M00110 C19/C18-Steroid hormone biosynthesis, and pregnenolone ≥ androstenedione ≥ estrone, showed no significant difference between the two groups. These pathway-level findings highlight fundamental dysregulation of polyamine biosynthesis, tryptophan catabolism, and steroid hormone metabolism in AR pangolins, which collectively may contribute to impaired reproductive function.

### 3.4 Combined analysis of microbial community and metabolites

**Figure 9:**
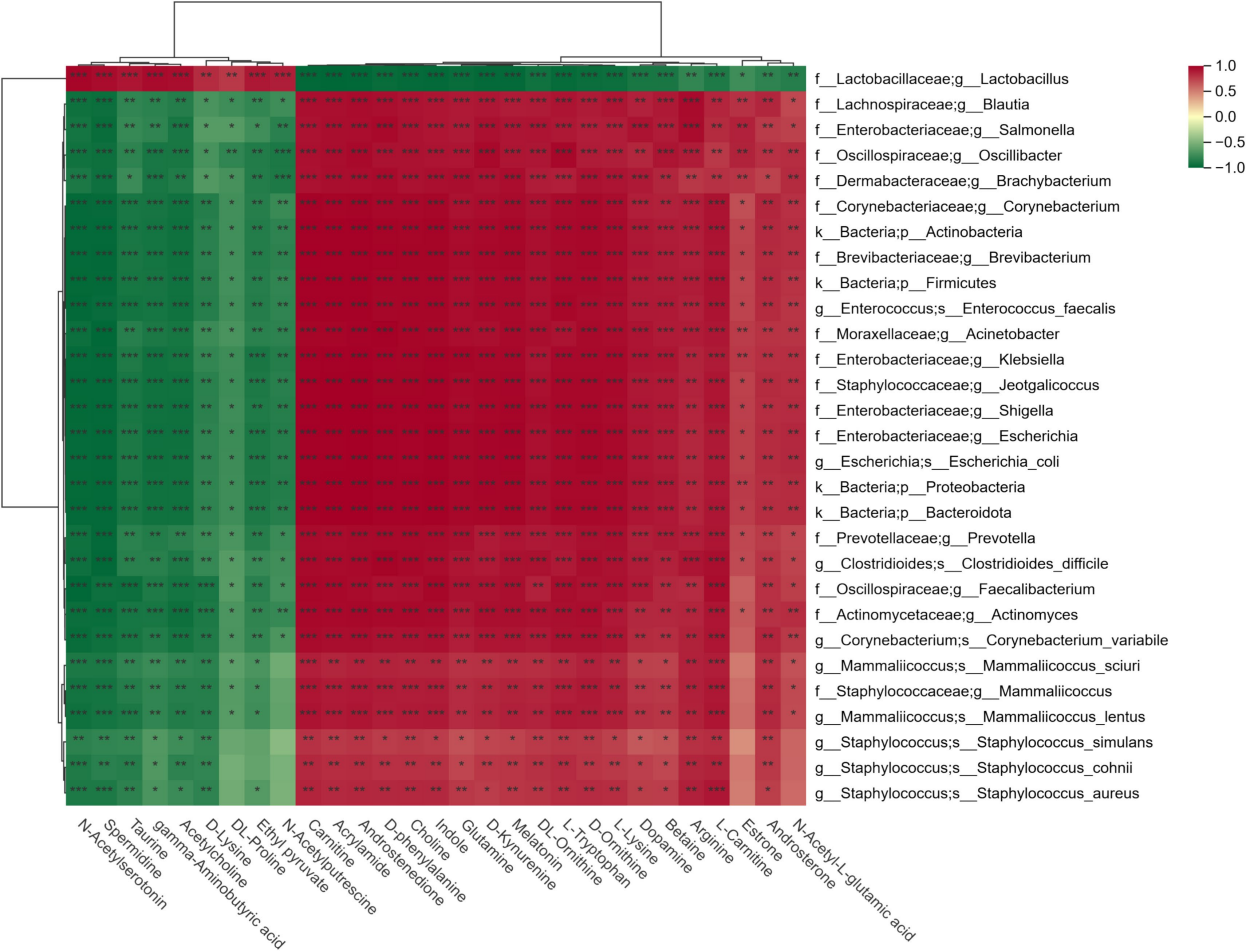
Correlation analysis. The correlation between differential microboota and metabolites. *p < 0.05, **p < 0.01, ***p < 0.001.

The microbial community showing a significant positive correlation with GABA is Lactobacillus; those showing a significant negative correlation include *E. coli*, *Staphylococcus aureus*, Klebsiella, Corynebacterium, Brevibacterium, Brachybacterium, Blautia, Prevotella, and Oscillibacter. Similarly, spermidine, putrescine, taurine, L-proline, and pyruvate show consistent correlations with the above microbial communities; acrylamide, glutamic acid and glutamine, lysine, and arginine show the exact opposite correlations with the above microbial communities (Figure 9).

## 4 Discussion

### 4.1 Effects of urine microbiota on host health

The alpha diversity of the urogenital microbiome is generally higher in symptomatic individuals than in asymptomatic controls (Robino et al., 2025). Higher alpha diversity of urine microbes has also been reported in patients with recurrent cystitis and bladder cancer (Sheng et al., 2025; Yoo et al., 2021). Under certain pathological conditions, microecological imbalance may lead to the co-occurrence of multiple bacteria, rather than being dominated by a relatively small number of healthy dominant bacteria. In sites such as the urinary tract/urinary system, a healthy state is often maintained by a low-diversity community dominated by specific dominant species (such as certain Lactobacillus species); an abnormally high diversity may indicate an imbalance or underlying inflammation/infection (Reasoner et al., 2025). High urinary microbial α diversity may be caused by the following factors: altered urethral barrier function, such as inflammation or injury that relaxes niche selection pressure (Reasoner et al., 2025); increased colonization of infectious or opportunistic pathogens (the presence of more than one bacterial community) (Robino et al., 2025).

The β diversity in the AR group was significantly different from that in the NR group. The urinary microbiota of pangolins with AR and those with NR changed significantly at the phylum and genus levels. Numerous studies have confirmed that dysbiosis of the urinary tract microbiota is associated with a variety of urinary system diseases (Fouts et al., 2012; Pearce et al., 2014; Shrestha et al., 2018; Siddiqui et al., 2012). This indicates that the urinary system possesses its own unique and relatively stable microecosystem; once the equilibrium of this system is disrupted, it may impact host health, immunity, and reproduction. In studies of human urine, Firmicutes is the most dominant phylum reported in the urine of healthy men and women (Perez-Carrasco et al., 2021). Actinobacteria participate in maintaining mucosal barrier homeostasis (Binda et al., 2018). Some studies believe that the higher the abundance of Actinobacteria, the better the semen quality. However, other studies have demonstrated a negative correlation of the abundance of Actinobacteria with testicular or semen health (Brandão et al., 2021; Contreras et al., 2023). In male urine samples, Proteobacteria are commonly found in patients with urogenital tract infections. These bacteria are typical urinary tract pathogens associated with urethritis, cystitis, or deeper urinary tract infections (Robino et al., 2025). Elevated relative abundance of Proteobacteria often indicates a disruption of the gut microbiota balance, a condition more common in patients with urinary tract diseases, those with chronic urinary symptoms, or immunocompromised individuals (Reasoner et al., 2025). Proteobacteria is the dominant phylum in many common diseases (Cao et al., 2023). Many Proteobacteria are pathogenic, serving as biomarkers for dysbiosis. Bacteroidetes are considered key players in maintaining the health and complex homeostasis of the microbiome (Gibiino et al., 2018). They are involved in immune regulation (i.e., activation of inflammation and autoimmune diseases), metabolic syndrome, and regulation of the gut–brain axis, with interesting therapeutic implications in mood disorders and neurological diseases (Gibiino et al., 2018). In contrast, men with leukocytospermia or prostatitis often exhibit an increased proportion of Bacteroidetes (Yao et al., 2022). As a cornerstone of the microbial system, Firmicutes was significantly more abundant in pangolins with NR, which may contribute to the ability of NR pangolins to maintain normal reproductive capacity. Actinobacteria exist in the urinary flora as part of resident or symbiotic bacteria. Their influence is more as part of the overall microbial community ecology than as a specific inflammatory inducer or defense factor. Proteobacteria may be associated with reproductive abnormalities. Bacteroidetes are also an important member of the microbiota, and their role in reproduction is not conclusive.

*Staphylococcus* spp. is considered a potential cause of male reproductive dysfunction (Okeleji et al., 2024). Staphylococcus is not only a commensal bacterium but also a major human pathogen causing widespread clinical infections (Shi et al., 2016). *S. aureus* can infect the male reproductive tract, causing conditions such as orchitis and epididymitis, and is a common pathogen causing urinary tract infections (UTIs) (Shi et al., 2016; Vasudevan, 2015). These infections can disrupt normal hormone secretion, ultimately impacting fertility (Shi et al., 2016; Vasudevan, 2015). *S. cohnii* strains isolated from mouse and human skin specimens have displayed significant preventive and therapeutic effects on *S. aureus*-mediated skin inflammation (Ito et al., 2021). Therefore, as the most abundant species in the genus *Staphylococcus*in this study (Supplementary Table S2), *S. cohnii* may play an important role in preventing infections caused by other bacteria including *S. aureus* in the urogenital tract.

Dietzia spp. is a potential pathogen (Koerner et al., 2009). Some species can even be used as probiotics to treat certain specific diseases (Menéndez et al., 2023; Oyanguren et al., 2024). As opportunistic pathogens, *Klebsiella* spp. mainly attack hospitalized immunocompromised patients and those with serious underlying diseases (Podschun and Ullmann, 1998). *Clostridioides* spp. (e.g., *C. difficile*), opportunistic intestinal pathogens, are one of the more common infectious agents of diarrhea (Pal et al., 2023). *Acinetobacter* spp. are pathogens (Alvanou et al., 2023; Bergogne-Bérézin, 2008). In this study, *Lactobacillus crispatus* was the most abundant *Lactobacillus* species in pangolin urine (Supplementary Table S2). Men whose semen was dominated by *L. crispatus* tended to have normal sperm morphology and concentration, with fewer white blood cells or anti-sperm IgA antibodies, all signs of better fertility (Grande et al., 2024). *Corynebacterium* spp. are mostly pathogenic bacteria (Metz et al., 2024; Möller et al., 2021; Tresch et al., 2023; Zendri et al., 2021). *Brevibacterium* spp. are opportunistic pathogens (Shweta et al., 2021). *Brachybacterium* spp. are opportunistic pathogens, but the probability of invasion is small (Tamai et al., 2018).

In this study, *E. coli* had the highest relative abundance among *Escherichia* spp. (Supplementary Table S2). *E. coli* is a common pathogen causing UTIs (Kudinha, 2017), leading to diseases such as orchitis and epididymitis (Bonner et al., 2021), which adversely affect male reproductive function. The presence of *E. coli* triggers an immune response, releasing inflammatory cytokines and reactive oxygen species (Mendoza-Chamizo et al., 2018). *E. coli* infection can also reduce testosterone levels by influencing the function of testicular interstitial cells (Danek, 2003), further reducing sexual desire, ultimately impacting fertility and reproductive performance.

*Jeotgalicoccus* spp. are harmful (Liang et al., 2024). *Shigella* infections can cause a strong systemic inflammatory response, leading to diseases such as orchitis or epididymitis, which can impact fertility (Marchiani et al., 2021; Su et al., 2021). *Shigella* infections can cause erectile or ejaculation problems in some cases (Zhao et al., 2020). *Salmonella* spp. can infect the urinary tract (Venyo, 2020). *Salmonella* infections can cause inflammation of the epididymis and testicles. A Holstein bull developed unilateral epididymo-orchitis associated with *Salmonella typhimurium*, resulting in testicular necrosis and potential impairment of fertility (Mahmoud et al., 2020).

From the perspective of microbial pathogenicity, these microbiota may be involved in reproductive system diseases in pangolins with AR

### 4.2 Effects of microbial functions and metabolites on host energy metabolism

The TCA cycle plays an important role in energy production, hormone synthesis, and sperm development and vitality (Frégeau-Proulx et al., 2022). The TCA cycle is accelerated to provide the body with more energy substrates (Rato et al., 2012). Pyruvate metabolism is the central node connecting glycolysis, mitochondrial energy production, and biosynthesis pathways. Its effects on male reproduction include energy supply, regulation of redox balance, biosynthetic precursors, and sperm function and maturation (Rotimi et al., 2024). Glycolysis breaks down glucose into pyruvate and produces ATP. Gluconeogenesis is essential for maintaining glucose homeostasis. The pathways of energy metabolism were improved in the AR group, but the levels of main functional substances such as pyruvate and glucose were still significantly lower than those in the NR group. Considering issues in energy absorption and supply in AR (energy dysfunction), the body is forced to compensatorily promote these metabolic pathways to supplement energy needs (Shimada et al., 2024; Zhao et al., 2023). Inadequate energy supply can hinder or even cease reproductive behavior (Martin et al., 2010). AR could be involved in underlying energy dysfunction, leading to compensatory enrichment of the aforementioned energy metabolism-related pathways, and ultimately affecting reproductive performance.

### 4.3 Effects of urine microbiota and metabolites on host psychology

Captivity can be a powerful chronic stressor for wild species (Lattin et al., 2012; Mason, 2010). Chronic stress from long-term captivity can lead to depression, weight loss, reproductive failure, reduced immune levels, and shorter lifespan (Birke, 2002; Islam et al., 2022). Excessive or long-term stress, especially chronic stress, can lead to not only metabolic disorders but also depression (Olff, 1999; Wang et al., 2024). Supplementing the diet of stressed male rats with GABA significantly reduced their anxiety-like behaviors (He et al., 2019). Supplementing the diet of Hanwoo steers under heat stress with GABA reduced their cortisol levels and the levels of stress-related markers (Barido et al., 2020). Patients with major depressive disorder (MDD) had significantly lower levels of spermidine (and putrescine), and spermidine (and putrescine) levels were negatively correlated with depression (Yazici et al., 2024). Taurine can reverse stress-induced neurotransmitter imbalances, increase levels of serotonin, dopamine, and norepinephrine, decrease corticosterone levels, prevent anhedonia (Wu et al., 2017), and has antidepressant effects (Zhu et al., 2023). L-proline can alleviate stress (Hamasu et al., 2009). Elevated levels of acrylamide metabolites in urine are associated with an increased risk of depressive symptoms (Wan et al., 2024). Chronic stress in mice resulted in decreased levels of glutamate and glutamine, accompanied by depressive-like behaviors (Son et al., 2018). DL-phenylalanine, a substance with antidepressant activity, was effective in treating depressed patients (Beckmann et al., 1977). A lack of lysine in the diet of rats led to increased anxiety under stress (Smriga et al., 2002), while supplementation with lysine and arginine reduced stress-induced anxiety and corticosterone release (Smriga and Torii, 2003). It is known that tryptophan levels are significantly reduced in patients with major depressive disorder (MDD) (Gu et al., 2021). Individuals with lower plasma pyruvate levels have a significantly increased risk of developing major depressive disorder (MDD) in the future; while individuals with higher plasma pyruvate levels have a more than 50% lower risk of developing depression (Radford-Smith et al., 2023). The above results indicate that the levels of potentially beneficial metabolites could associated with psychological state were significantly higher in the NR group than in the AR group. NR group might also be associated with depressive tendencies due to chronic stress. The microbiota is positively or negatively correlated with these metabolic processes, and it has also been reported to have effects on stress, anxiety, and depression. Moreover, *E. coli* infection can disrupt the microbial gut–brain axis, with infected mice showing significantly increased anxiety- and depressive-like behaviors (Rondepierre et al., 2024). *S. aureus* can cause depressive-like behaviors by producing staphylococcal enterotoxins that damage the nervous system; treatment to reduce the abundance of *S. aureus* can improve the behavior of mice with depression (Qiu et al., 2023). The abundance of *Klebsiella* spp. is significantly higher in patients with major depressive disorder than in healthy controls (Lin et al., 2017). *Lactobacillus* spp. in the intestine are thought to have beneficial effects on stress responses and depressive disorders (Aizawa et al., 2016). The abundance of *Corynebacterium* spp. decreased whereas the abundance of beneficial bacteria, especially *Lactobacillus* spp., increased after antidepressant treatment (Lv et al., 2021). *Brevibacterium* spp. and *Brachybacterium* spp. were detected in rats with chronic unpredictable mild stress (CUMS)-induced depression (Lv et al., 2021). The analysis at the genus level indicated an elevated abundance of *Blautia* with decreased abundance of *Prevotella* in patients with major depression (Socała et al., 2021). The abundance of *Oscillibacter* also significantly increased in patients with major depressive disorder (MDD) (Jiang et al., 2015). From the perspective of microbial depression, pangolins in the AR group could be involved in a higher risk of underlying depression, but pangolins in the NR group might also associated with a risk of underlying depression due to long-term chronic stress.

### 4.4 Effects of urine microbiota and metabolites on host reproduction

#### 4.4.1 Microbiota

*Actinomyces* spp. (belonging to the phylum Actinobacteria) were negatively correlated with erectile function and may act as key pathogenic contributors (Kaur and Prabha, 2014). The most abundant genera found in patients with erectile dysfunction (ED) included *Blautia*, *Faecalibacterium*, *Escherichia*, and *Shigella* (Kang et al., 2023). Among patients with ED, 23.7% had bacterial prostatitis; the most common isolate was *E. coli*, followed by *S. aureus* (El-Kamel et al., 2024). *E. faecalis* interfered with male fertility (Farsimadan and Motamedifar, 2020; Shahroodian et al., 2022). These pathogens might affect sexual function and cause inflammation, thus contributing directly or indirectly to reproductive abnormalities in male pangolins.

#### 4.4.2 GnRH–FSH/LH–testosterone axis

The GnRH secretion (KO04929) was significantly enriched in the NR group. The pulsatile release of GnRH in the hypothalamus stimulates the anterior pituitary to release FSH and LH. In men, normal FSH and LH levels are responsible for inducing spermatogenesis and maintaining high levels of testosterone in the testes (Conn and Crowley, 1991). GnRH can induce androgen production and spermatogenesis and improve sexual function (Dabaja and Schlegel, 2014; Mortimer et al., 1974). Its deficiency can lead to gonadotropin-induced hypogonadism, thus causing male infertility (Silveira et al., 2002). The AR group showed a significant reduction in GnRH secretion, which could be associated with their inability to mate and reproduce normally.

Studies have observed that L-carnitine has a positive effect on reproductive hormones. In male rats subjected to lead-induced reproductive toxicity, L-carnitine supplementation increased serum levels of follicle-stimulating hormone (FSH), luteinizing hormone (LH), and testosterone (Abdel-Emam and Ahmed, 2021). Dopamine (DA) is one of the most crucial neurotransmitters regulating male reproductive behavior and function. It not only participates in the formation of libido and courtship motivation, but also directly affects mating behavior, penile erection, ejaculation, testosterone regulation, and the function of the hypothalamus-pituitary-gonadal (HPG) axis (Hull et al., 2004; Melis et al., 2022). Intracavernosal injection of acetylcholine (ACh) in monkeys can induce penile erection (Stief et al., 1989). ACh acts on the medial preoptic area and nucleus accumbens, and regulates various stages of sexual intercourse—including mating, penile penetration, and ejaculation—by coordinating signal transduction between ACh and dopamine (Floody, 2014). The levels of these hormones increased in male rats of different ages after taurine administration, indicating enhancement of endocrine function (Li et al., 2023; Yang et al., 2010). In a model of reproductive dysfunction, taurine increased the levels of GnRH and FSH, indicating its stimulating effect on the HPG axis (Yang et al., 2013). In aged male rats, taurine supplementation was associated with improved sexual behavior, including increased mating frequency and shortened ejaculation latency (Yang et al., 2013). The enhancement of these behaviors was related to the stimulating effects of taurine on testosterone production and nitric oxide (NO) synthesis, both of which are essential for sexual function (Yang et al., 2013). Dietary choline supplementation maintained and promoted the reproductive performance of bulls (Johnson et al., 2010; Prasetiyono et al., 2020). Betaine can effectively restore testosterone production reduced by heat stress and plays an important role in stabilizing the reproductive endocrine axis (Xiong et al., 2023). The above metabolites were significantly enriched in the NR group, which might be involved in their positive effects on reproductive function.

#### 4.4.3 Polyamine and amino acid metabolism

Studies on giant pandas speculated that the decline in male giant panda reproductive capacity was related to an imbalance in arginine synthesis (Zhang et al., 2022). Studies have shown that L-arginine can enhance sexual performance (Rhim et al., 2019; Tahvilian et al., 2024). However, L-arginine deficiency has been reported to reduce sex hormone levels in captive giant pandas because decreased levels of its metabolites (including nitric oxides and polyamines) influence the expression of sexual desire and sexual behavior during mate selection (Zhang et al., 2022). In this study, our findings suggest that reproductive performance might be influenced not only by arginine synthesis itself but also by broader alterations in arginine metabolism.

Polyamine biosynthesis, arginine ≥ agmatine ≥ putrescine ≥ spermidine (M00133): The urinary spermidine content in NR was significantly higher than that in AR. Studies showed that spermidine promoted spermatogenic cell proliferation in mice with diabetes by activating glycolysis (Wang et al., 2022). Spermidine can improve testicular dysfunction caused by triptolide; protect germ cell development, testosterone synthesis, and oxidative stress balance; reduce inflammation (Zhao et al., 2021); and extend reproductive lifespan (Wu et al., 2023).

Polyamine biosynthesis, arginine ≥ ornithine ≥ putrescine (M00134): The level of *N*-acetylputrescine was significantly higher in the NR group than in the AR group. Putrescine can enhance piglet mucosal immune function and inhibit chronic inflammation (Mumcu et al., 2020). Patients with oligoasthenoteratozoospermia had lower levels of arginine, putrescine, and spermine than controls (Mumcu et al., 2020). Arginine supplementation increased testicular arginine and putrescine levels, thereby improving testicular development and semen quality in boars (Wei et al., 2022). Putrescine improved the decline in semen quality and testosterone levels in boars caused by heat stress (Yu et al., 2025). Although the arginine level in the AR group was significantly higher than that in the NR group, the results indicate that the arginine metabolic utilization efficiency in the AR group could be lower than that in the NR group, which might be associated with the poor reproductive performance.

Tryptophan metabolism, tryptophan ≥ kynurenine ≥ 2-aminomuconate (M00038): High levels of kynurenine activate the aryl hydrocarbon receptor (AhR), inhibiting the expression of cholesterol side-chain cleavage enzyme (CYP11A1) and 17α-hydroxylase (CYP17A1) in Leydig cells, thereby reducing the activity of enzymes involved in the key steps of testosterone synthesis (Shen et al., 2023). Kynurenine accumulated in the brain activates the AhR signaling pathway, inhibiting the release of GnRH from the hypothalamus, leading to a decrease in pituitary LH secretion, and further reducing testosterone synthesis (Shen et al., 2023). Overall, the two tryptophan metabolic pathways might not be associated with beneficial to animal reproduction. Tryptophan levels were significantly higher in the AR group, which could be involved in their poor reproductive performance.

Tryptophan is metabolized to NAS, which can reverse dopamine depletion and improve motor behavior (Aguiar et al., 2005). Under certain stress conditions, NAS stimulates tropomyosin-related kinase receptor B (TrkB) receptor-mediated neurogenesis, which plays an antioxidant and anti-apoptotic role in oxidative stress–induced neurotoxicity (Yoo et al., 2017). Tryptophan is metabolized to indole, and indole production in male rodents under chronic stress can enhance mood/anxiety and increase adrenal medullary activity (Mir et al., 2020). Analysis of these metabolites suggests that the stress and stress state of the NR group might be more favorable than that of the AR group.

#### 4.4.4 Sex hormone metabolism

Androsterone, testosterone, dehydroepiandrosterone, estrone, and androstenedione were significantly enriched in the AR group. The metabolic pathways were C19-Steroid hormone biosynthesis (androgen backdoor pathway), pregnenolone ≥ androsterone ≥ dihydrotestosterone (M00977), C19-Steroid hormone biosynthesis, pregnenolone ≥ testosterone ≥ dihydrotestosterone (M00976), C19/C18-Steroid hormone biosynthesis, and pregnenolone ≥ androstenedione ≥ estrone (M00110). Reduced androsterone levels are closely related to decreased sexual desire (Bloch et al., 2006), and testosterone enhances the sexual desire of hypogonadal men (Boloña et al., 2007). Increased levels of total estrogen may lead to decreased sexual desire (Regan, 1999). Both androsterone and testosterone are metabolized to dihydrotestosterone. However, no significant difference in dihydrotestosterone levels was found between the two groups. The androgen and testosterone levels in the AR group could be involved in metabolic disorders, leading to impaired function. Meanwhile, the significantly elevated estrogen levels might also contribute to fertility.

#### 4.4.5 Neurotransmitter regulation

Melatonin biosynthesis, animals, tryptophan ≥ serotonin ≥ melatonin (M00037): Tryptophan is metabolized into serotonin, which, on the one hand, can improve the mental state of animals, but, on the other hand, can inhibit sexual arousal and motivation. Serotonin usually inhibits male sexual behavior (Bitran et al., 1987; Hull et al., 2004). As a neurotransmitter, serotonin regulates mood and appetite while significantly inhibiting the sexual desire and mating behavior of male animals (Xie et al., 2025). Serotonin is further metabolized into melatonin. Evidence shows that multiple, high-dose injections of melatonin in male rats suppressed sexual function. In contrast, systemic injections of low doses (10–100 μg/kg) of the hormone stimulated sexual activity in normal male rats (Drago, 2000). Melatonin treatment improved erectile function and alleviated neuropathy and fibrosis in rats with diabetes. These changes might be related to reduced oxidative stress, inhibition of the p38MAPK signaling pathway, and alleviation of neuropathy (Zhang et al., 2018).

Serotonergic, GABAergic, and dopaminergic neurotransmission (Clarke et al., 2013; González-Arancibia et al., 2019; Lenschow and Lima, 2020; Mayer et al., 2015) are considered to be closely related to sexual desire (Li et al., 2021). Cholinergic agonists and antagonists reduce sexual activity, and cholinergic drugs can reverse erectile and ejaculatory difficulties (Hull et al., 1988; Meston and Frohlich, 2000). Cholinergic inhibition leads to suppressed mating activity (Iovino et al., 2019). Environmental stressors and psychological factors can impact GABA metabolism. Studies on captive male giant pandas have reported an impact of environmental restrictions on the synthesis of neurotransmitters, including GABA, leading to reduced sexual desire and mating behavior (Zhang et al., 2022). Analysis of seceral metabolic pathways suggests that sexual desire might be lower in the AR group than in the NR group, and chronic stress could be associated with a greater impact. This might contribute to the superior reproductive performance of the NR group compared to the AR group.

### 4.5 Study limitations and future perspectives

Although we collected samples every 2 h, moisture evaporation and the reduction or even disappearance of volatile substances were inevitable, which affected the types and concentrations of metabolites to some extent. Due to the lack of direct research on pangolins, some inferences are based on other mammals, which may affect the accuracy of these conclusions. Furthermore, our sample size is relatively small (our captive population is small), and there is currently a lack of validation studies on pangolin urinary proteomics/transcriptomics, which also affects the accuracy of these conclusions. Despite these limitations, this study lays the foundation for future research and emphasizes the need for more in-depth validation studies to further explore the impact of captivity on pangolin reproduction.

Implications of this study for conservation and management: The results indicate that the microbiome and metabolites may be important factors influencing the reproduction of male Malayan pangolins. Therefore, future research may attempt to verify the effects of adding probiotics and certain key metabolites on male reproduction, which may have beneficial effects on pangolin reproduction and conservation.

## 5 Conclusion

In summary, this study revealed a series of significant differences in the AR group: (1) The structure of potentially beneficial and harmful in the gut microbiota of the AR group was significantly altered, with an increased abundance of harmful bacteria (such as Proteobacteria, *Shigella* spp., *Salmonella* spp., *E. coli*, and *E. faecalis*, etc.), which might contribute to inflammation and depression, and some microbiomes could even be associated with factors that are detrimental to reproduction. However, this association needs to be further verified in larger samples and dynamic studies. (2) Metabolomics analysis of the AR group showed enrichment of energy metabolism pathways (such as the TCA cycle and pyruvate metabolism), but decreased levels of related substrates (such as pyruvate), exhibiting a phenomenon of high enrichment but insufficient substrates, which might suggest that there could be an imbalance in energy metabolism. This result needs to be further studied through precise measurement of energy substances and functional experiments. (3) Androgen metabolism and signaling pathways were significantly disordered in the AR group: GnRH secretion secretion was reduced in the AR group, and the metabolic efficiency of androsterone and testosterone was relatively low. These physiological hypotheses require further verification through hormone level and behavioral experiments. (4) The AR group might be associated with arginine metabolism disorders. However, the correlation and causal mechanism are still unclear and require further functional studies to confirm. These findings highlight the special changes in the AR group at the level of microbial community and host metabolism, providing a new perspective for the study of the mechanism of male reproductive abnormalities; at the same time, due to the limitations of sample size and conditions, all conclusions need to be further verified and supplemented in more studies.

## Data Availability

The datasets presented in this study can be found in online repositories. The names of the repository/repositories and accession number(s) can be found at: https://www.ncbi.nlm.nih.gov/, PRJNA1286757.

## Footnotes

1
https://www.r-project.org

2
http://www.kegg.jp/kegg/compound/

3
http://www.kegg.jp/kegg/pathway.html

## References

[ref1] Abdel-Emam R. A. Ahmed E. A. (2021). Ameliorative effect of L-carnitine on chronic lead-induced reproductive toxicity in male rats. Vet. Med. Sci. 7, 1426–1435. doi: 10.1002/vms3.473, 33724722 PMC8294385

[ref2] Aguiar L. M. Macedo D. S. de Freitas R. M. de Albuquerque Oliveira A. Vasconcelos S. M. M. de Sousa F. C. F. . (2005). Protective effects of N-acetylserotonin against 6-hydroxydopamine-induced neurotoxicity. Life Sci. 76, 2193–2202. doi: 10.1016/j.lfs.2004.09.035, 15733934

[ref3] Aizawa E. Tsuji H. Asahara T. Takahashi T. Teraishi T. Yoshida S. . (2016). Possible association of *Bifidobacterium* and *Lactobacillus* in the gut microbiota of patients with major depressive disorder. J. Affect. Disord. 202, 254–257. doi: 10.1016/j.jad.2016.05.038, 27288567

[ref4] Alvanou M. V. Feidantsis K. Staikou A. Apostolidis A. P. Michaelidis B. Giantsis I. A. (2023). Probiotics, prebiotics, and synbiotics utilization in crayfish aquaculture and factors affecting gut microbiota. Microorganisms 11:1232. doi: 10.3390/microorganisms11051232, 37317206 PMC10220723

[ref5] Arora B. Pei K. J. Chin S. C. (2023). New horizons in the reproductive biology of Chinese pangolin (*Manis pentadactyla*) using the gonadal hormonal profile. Sci. Rep. 13:16630. doi: 10.1038/s41598-023-43237-0, 37789068 PMC10547839

[ref6] Barido F. H. Lee C. W. Park Y. S. Kim D. Y. Lee S. K. (2020). The effect of a finishing diet supplemented with γ-aminobutyric acids on carcass characteristics and meat quality of Hanwoo steers. Anim. Biosci. 34, 621–632. doi: 10.5713/ajas.20.0224, 32882778 PMC7961294

[ref7] Beckmann H. Strauss M. Ludolph E. (1977). Dl-phenylalanine in depressed patients: an open study. J. Neural Transm. 41, 123–134. doi: 10.1007/BF01670277, 335027

[ref8] Belizário J. E. Napolitano M. (2015). Human microbiomes and their roles in dysbiosis, common diseases, and novel therapeutic approaches. Front. Microbiol. 6:1050. doi: 10.3389/fmicb.2015.01050, 26500616 PMC4594012

[ref9] Bergogne-Bérézin E. (2008). “Importance of Acinetobacter spp.,” in Acinetobacter Biology and Pathogenesis. Infectious Agents and Pathogenesis, eds. Mauro Bendinelli H. F. Bergogne-Bérézin E. (New York, NY: Springer), 1–18.

[ref10] Binda C. Lopetuso L. R. Rizzatti G. Gibiino G. Cennamo V. Gasbarrini A. (2018). Actinobacteria: a relevant minority for the maintenance of gut homeostasis. Dig. Liver Dis. 50, 421–428. doi: 10.1016/j.dld.2018.02.012, 29567414

[ref11] Birke L. (2002). Effects of browse, human visitors and noise on the behaviour of captive orang utans. Anim. Welf. 11, 189–202. doi: 10.1017/S0962728600028141

[ref12] Bitran D. Hull E. M. J. N. Reviews B. (1987). Pharmacological analysis of male rat sexual behavior. Neurosci. Biobehav. Rev. 11, 365–389. doi: 10.1016/S0149-7634(87)80008-8 2830564

[ref13] Bloch M. Rubinow D. R. Berlin K. Kevala K. R. Kim H.-Y. Schmidt P. J. (2006). Monoamines and neurosteroids in sexual function during induced hypogonadism in healthy men. Arch. Gen. Psychiatry 63, 450–456. doi: 10.1001/archpsyc.63.4.450, 16585475

[ref14] Boloña E. R. Uraga M. V. Haddad R. M. Tracz M. J. Sideras K. Kennedy C. C. . (2007). Testosterone use in men with sexual dysfunction: a systematic review and meta-analysis of randomized placebo-controlled trials. Mayo Clin. Proc. 82, 20–28. doi: 10.4065/82.1.20, 17285782

[ref15] Bonner M. Sheele J. M. Cantillo-Campos S. Elkins J. M. (2021). A descriptive analysis of men diagnosed with epididymitis, orchitis, or both in the emergency department. Cureus 13:e15800. doi: 10.7759/cureus.15800, 34306868 PMC8294204

[ref16] Brandão P. Gonçalves-Henriques M. Ceschin N. (2021). Seminal and testicular microbiome and male fertility: a systematic review. Porto Biomed. J. 6:e151. doi: 10.1097/j.pbj.0000000000000151, 34881355 PMC8647872

[ref17] Buchfink B. Xie C. Huson D. H. (2015). Fast and sensitive protein alignment using DIAMOND. Nat. Methods 12, 59–60. doi: 10.1038/nmeth.3176, 25402007

[ref18] Cao Y. Wang H. Jin Z. Hang J. Jiang H. Wu H. . (2023). Characterization of non-obstructive azoospermia in men using gut microbial profiling. J. Clin. Med. 12:701. doi: 10.3390/jcm12020701, 36675631 PMC9861525

[ref19] Cen P. Sun J. Wang Q. Zhang F. Mo L. Mahmood A. . (2023). Only Sunda and Chinese pangolin (Pholidota) are naturally distributed in China. Integr. Zool. 18, 704–709. doi: 10.1111/1749-4877.12697, 36519580

[ref20] Challender D. Willcox D. H. A. Panjang E. Lim N. Nash H. Heinrich S. . (2019). Sunda pangolin (*Manis javanica*). The IUCN Red List of Threatened Species 2019, e.T12763A123584856. doi: 10.2305/IUCN.UK.2019-3.RLTS.T12763A123584856.en

[ref21] Chang Y. Yu J. Wang T. Chin S. Wei Y. Chen T. . (2020). Investigation of epididymal proteins and general sperm membrane characteristics of Formosan pangolin (*Manis pentadactyla pentadactyla*). BMC Zool. 5:15. doi: 10.1186/s40850-020-00064-4

[ref22] Chin S. C. Lien C. Y. Chan Y. T. Chen C. L. Yang Y. C. Yeh L. S. (2012). Monitoring the gestation period of rescued Formosan pangolin (*Manis pentadactyla pentadactyla*) with progesterone radioimmunoassay. Zoo Biol. 31, 479–489. doi: 10.1002/zoo.20413, 21866570

[ref23] Chong J. L. Panjang E. Willcox D. Nash H. C. Semiadi G. Sodsai W. . (2020). “Sunda pangolin *Manis javanica* (Desmarest, 1822),” in Pangolins, eds. Challender N. H. Waterman C. (London: Academic Press), 89–108.

[ref24] Clarke G. Grenham S. Scully P. Fitzgerald P. Moloney R. Shanahan F. . (2013). The microbiome-gut-brain axis during early life regulates the hippocampal serotonergic system in a sex-dependent manner. Mol. Psychiatry 18, 666–673. doi: 10.1038/mp.2012.77, 22688187

[ref25] Collino S. Martin F. P. Rezzi S. (2013). Clinical metabolomics paves the way towards future healthcare strategies. Br. J. Clin. Pharmacol. 75, 619–629. doi: 10.1111/j.1365-2125.2012.04216.x, 22348240 PMC3575929

[ref26] Conn P. M. Crowley W. F. Jr. (1991). Gonadotropin-releasing hormone and its analogues. N. Engl. J. Med. 324, 93–103. doi: 10.1056/NEJM199101103240205 1984190

[ref27] Contreras M. J. Núñez-Montero K. Bruna P. Zárate A. Pezo F. García M. . (2023). Mammals’ sperm microbiome: current knowledge, challenges, and perspectives on metagenomics of seminal samples. Front. Microbiol. 14:1167763. doi: 10.3389/fmicb.2023.1167763, 37138598 PMC10149849

[ref28] Czekala N. M. Hodges J. K. Gause G. E. Lasley B. L. (1980). Annual circulating testosterone levels in captive and free-ranging male armadillos (*Dasypus novemcinctus*). J. Reprod. Fertil. 59, 199–204. doi: 10.1530/jrf.0.0590199, 6249926

[ref29] Dabaja A. A. Schlegel P. N. (2014). Medical treatment of male infertility. Transl. Andrology Urol. 3, 9–16. doi: 10.3978/j.issn.2223-4683.2014.01.06, 26816749 PMC4708300

[ref30] Dai Z. Xie B. Xie C. Xiang J. Wang X. Li J. . (2024). Comparative metagenomic analysis of the gut microbiota of captive pangolins: a case study of two species. Animals 15:57. doi: 10.3390/ani15010057, 39795000 PMC11718824

[ref31] Danek J. (2003). Effect of *Escherichia coli* endotoxin on the levels of testosterone and estradiol-17β in blood serum, seminal plasma and semen characteristics of stallion. Bull. Vet. Inst. Pulawy 47, 191–201.

[ref32] de Moura F. B. C. Lacerda Z. A. Catão-Dias J. L. Navas-Suárez P. E. Werther K. Simões S. R. J. S. . (2024). Background and common lesions in the female reproductive organs of giant anteaters (*Myrmecophaga tridactyla*). Front. Vet. Sci. 11:1287872. doi: 10.3389/fvets.2024.1287872, 38328261 PMC10847298

[ref33] Drago F. (2000). Acute low doses of melatonin restore full sexual activity in impotent male rats. Brain Res. 878, 98–104. doi: 10.1016/S0006-8993(00)02715-3, 10996140

[ref34] El-Kamel M. F. Elhanbly S. M. Abbas E. H. Masallat D. T. (2024). Prevalence and microbiological characteristics of bacterial prostatitis in patients with erectile dysfunction. Egypt. J. Med. Microbiol. 33, 123–129. doi: 10.21608/ejmm.2024.281511.1240

[ref35] Fallon L. Browning G. R. Felton R. Belmer M. Riveraluis X. Durrant B. . (2026). Female reproductive physiology in the aardvark (*Orycteropus afer*): a novel case report. Theriogenol. Wild 8:100145. doi: 10.1016/j.therwi.2026.100145

[ref36] Farsimadan M. Motamedifar M. (2020). Bacterial infection of the male reproductive system causing infertility. J. Reprod. Immunol. 142:103183. doi: 10.1016/j.jri.2020.103183, 32853846

[ref37] Floody O. R. (2014). Role of acetylcholine in control of sexual behavior of male and female mammals. Pharmacol. Biochem. Behav. 120, 50–56. doi: 10.1016/j.pbb.2014.02.007, 24561063

[ref38] Fouts D. E. Pieper R. Szpakowski S. Pohl H. Knoblach S. Suh M. J. . (2012). Integrated next-generation sequencing of 16S rDNA and metaproteomics differentiate the healthy urine microbiome from asymptomatic bacteriuria in neuropathic bladder associated with spinal cord injury. J. Transl. Med. 10:174. doi: 10.1186/1479-5876-10-174, 22929533 PMC3511201

[ref39] Frégeau-Proulx L. Lacouture A. Berthiaume L. Weidmann C. Harvey M. Gonthier K. . (2022). Multiple metabolic pathways fuel the truncated tricarboxylic acid cycle of the prostate to sustain constant citrate production and secretion. Mol. Metab. 62:101516. doi: 10.1016/j.molmet.2022.101516, 35598879 PMC9168698

[ref40] Fromme L. Yogui D. R. Alves M. H. Desbiez A. L. J. Langeheine M. Santos A. L. Q. . (2023). Spermatogenesis in the giant anteater (*Myrmecophaga tridactyla*). Theriogenol. Wild 2:100018. doi: 10.1016/j.therwi.2023.100018

[ref41] Gibiino G. Lopetuso L. R. Scaldaferri F. Rizzatti G. Binda C. Gasbarrini A. (2018). Exploring Bacteroidetes: metabolic key points and immunological tricks of our gut commensals. Dig. Liver Dis. 50, 635–639. doi: 10.1016/j.dld.2018.03.016, 29650468

[ref42] González-Abarzúa M. Lim J. Panjang E. Ng K. C. H. Goossens B. Sipangkui S. . (2025). Maternal den preferences, reproductive behavior, and spatial use of a wild *Manis javanica* during maternal and non-maternal stages in Sabah. Borneo. Endanger. Species Res. 58, 223–240. doi: 10.3354/esr01443

[ref43] González-Arancibia C. Urrutia-Piñones J. Illanes-González J. Martinez-Pinto J. Sotomayor-Zárate R. Julio-Pieper M. . (2019). Do your gut microbes affect your brain dopamine? Psychopharmacology (Berl) 236, 1611–1622. doi: 10.1007/s00213-019-05265-5, 31098656

[ref44] Grande G. Graziani A. De Toni L. Garolla A. Ferlin A. (2024). Male tract microbiota and male infertility. Cells 13:1275. doi: 10.3390/cells13151275, 39120306 PMC11312145

[ref45] Gray R. J. Van Le D. Nguyen H. T. T. Cau L. N. Van Nguyen T. Van Pham T. . (2023). Home ranges and activity patterns of Sunda pangolins *Manis javanica* (Pholidota: Manidae) in Vietnam. J. Asia-Pac. Biodivers. 16, 421–431. doi: 10.1016/j.japb.2023.05.005

[ref46] Gu X. Ke S. Wang Q. Zhuang T. Xia C. Xu Y. . (2021). Energy metabolism in major depressive disorder: recent advances from omics technologies and imaging. Br. J. Anaesth. 141:111869. doi: 10.1016/j.biopha.2021.111869, 34225015

[ref47] Hamasu K. Haraguchi T. Kabuki Y. Adachi N. Tomonaga S. Sato H. . (2009). L-proline is a sedative regulator of acute stress in the brain of neonatal chicks. Amino Acids 37, 377–382. doi: 10.1007/s00726-008-0164-0, 18696178

[ref48] He Y. Ouyang J. Hu Z. Yang J. Chu Y. Huang S. . (2019). Intervention mechanism of repeated oral GABA administration on anxiety-like behaviors induced by emotional stress in rats. Psychiatry Res. 271, 649–657. doi: 10.1016/j.psychres.2018.12.025, 30791338

[ref49] Howell-Stephens J. Bernier D. Brown J. S. Mulkerin D. Santymire R. M. (2013). Using non-invasive methods to characterize gonadal hormonal patterns of southern three-banded armadillos (*Tolypeutes matacus*) housed in north American zoos. Anim. Reprod. Sci. 138, 314–323. doi: 10.1016/j.anireprosci.2013.02.004, 23541612

[ref50] Hua L. Gong S. Wang F. Li W. Ge Y. Li X. . (2015). Captive breeding of pangolins: current status, problems and future prospects. Zoo Keys 507, 99–114. doi: 10.3897/zookeys.507.6970, 26155072 PMC4490220

[ref52] Hull E. M. Bitran D. Pehek E. A. Holmes G. M. Warner R. K. Band L. C. . (1988). Brain localization of cholinergic influence on male sex behavior in rats: agonists. Pharmacol. Biochem. Behav. 31, 175–178. doi: 10.1016/0091-3057(88)90329-2 3252248

[ref53] Hull E. M. Muschamp J. W. Sato S. (2004). Dopamine and serotonin: influences on male sexual behavior. Physiol. Behav. 83, 291–307. doi: 10.1016/j.physbeh.2004.08.018, 15488546

[ref54] Huson D. H. Mitra S. Ruscheweyh H.-J. Weber N. Schuster S. C. (2011). Integrative analysis of environmental sequences using MEGAN4. Genome Res. 21, 1552–1560. doi: 10.1101/gr.120618.111, 21690186 PMC3166839

[ref55] Iovino M. Messana T. Iovino E. De Pergola G. Guastamacchia E. Giagulli V. A. . (2019). Neuroendocrine mechanisms involved in male sexual and emotional behavior. Endocr. Metab. Immune Disord. Drug Targets 19, 472–480. doi: 10.2174/1871530319666190131155310, 30706797 PMC7360913

[ref56] Islam P. Saha P. Khatun P. Hossain M. I. Islam A. Sachi S. (2022). Stress biomarker: a review on glucocorticoids concentration pattern & its impact on captive animals. J. Fish. 2, 88–100. doi: 10.18801/jflvs.020222.10

[ref57] Ito Y. Sasaki T. Li Y. Tanoue T. Sugiura Y. Skelly A. N. . (2021). *Staphylococcus cohnii* is a potentially biotherapeutic skin commensal alleviating skin inflammation. Cell Rep. 35:109052. doi: 10.1016/j.celrep.2021.109052, 33910010

[ref58] Jiang H. Ling Z. Zhang Y. Mao H. Ma Z. Yin Y. . (2015). Altered fecal microbiota composition in patients with major depressive disorder. Brain Behav. Immun. 48, 186–194. doi: 10.1016/j.bbi.2015.03.016, 25882912

[ref59] Jiao W. Liu L. Zeng Z. Li L. Chen J. (2022). Differences in gut microbes in captive pangolins and the effects of captive breeding. Front. Microbiol. 13:1053925. doi: 10.3389/fmicb.2022.1053925, 36560954 PMC9763570

[ref60] Johnson A. R. Craciunescu C. N. Guo Z. Teng Y.-W. Thresher R. J. Blusztajn J. K. . (2010). Deletion of murine choline dehydrogenase results in diminished sperm motility. FASEB J. 24, 2752–2761. doi: 10.1096/fj.09-153718, 20371614 PMC2909292

[ref61] Johnston S. D. Nicolson V. Madden C. Logie S. Pyne M. Roser A. . (2007). Assessment of reproductive status in male echidnas. Anim. Reprod. Sci. 97, 114–127. doi: 10.1016/j.anireprosci.2005.12.016, 16476529

[ref62] Kanehisa M. Furumichi M. Tanabe M. Sato Y. Morishima K. (2017). KEGG: new perspectives on genomes, pathways, diseases and drugs. Nucleic Acids Res. 45, D353–D361. doi: 10.1093/nar/gkw1092, 27899662 PMC5210567

[ref63] Kang J. Wang Q. Wang S. Pan Y. Niu S. Li X. . (2023). Characteristics of gut microbiota in patients with erectile dysfunction: a Chinese pilot study. World J. Mens Health 42:363. doi: 10.5534/wjmh.220278 37382280 PMC10949016

[ref64] Karlsson F. H. Fåk F. Nookaew I. Tremaroli V. Fagerberg B. Petranovic D. . (2012). Symptomatic atherosclerosis is associated with an altered gut metagenome. Nat. Commun. 3:1245. doi: 10.1038/ncomms2266, 23212374 PMC3538954

[ref65] Kaur K. Prabha V. (2014). Spermagglutinating Escherichia coli and its role in infertility: *in vivo* study. Microb. Pathog. 69-70, 33–38. doi: 10.1016/j.micpath.2014.03.010, 24685696

[ref66] Khamis M. M. Adamko D. J. El-Aneed A. (2017). Mass spectrometric based approaches in urine metabolomics and biomarker discovery. Mass Spectrom. Rev. 36, 115–134. doi: 10.1002/mas.21455, 25881008

[ref67] Knott K. K. Roberts B. M. Maly M. A. Vance C. K. Debeachaump J. Majors J. . (2013). Fecal estrogen, progestagen and glucocorticoid metabolites during the estrous cycle and pregnancy in the giant anteater (*Myrmecophaga tridactyla*): evidence for delayed implantation. Reprod. Biol. Endocrinol. 11:83. doi: 10.1186/1477-7827-11-83, 23981950 PMC3765926

[ref68] Koerner R. J. Goodfellow M. Jones A. L. (2009). The genus Dietzia: a new home for some known and emerging opportunist pathogens. FEMS Immunol. Med. Microbiol. 55, 296–305. doi: 10.1111/j.1574-695X.2008.00513.x, 19159434

[ref69] Kudinha T. (2017). “The pathogenesis of *Escherichia coli* urinary tract infection,” in *Escherichia coli* - Recent Advances on Physiology, Pathogenesis and Biotechnological Applications, ed. Samie A. (London: Intech).

[ref70] Lattin C. R. Bauer C. M. de Bruijn R. Romero L. M. (2012). Hypothalamus–pituitary–adrenal axis activity and the subsequent response to chronic stress differ depending upon life history stage. Gen. Comp. Endocrinol. 178, 494–501. doi: 10.1016/j.ygcen.2012.07.013, 22841762

[ref71] Lenschow C. Lima S. Q. (2020). In the mood for sex: neural circuits for reproduction. Curr. Opin. Neurobiol. 60, 155–168. doi: 10.1016/j.conb.2019.12.001, 31901622

[ref72] Li Y. Hua Y. Xiang Z. Xu X. Zhang S. Wang X. . (2024). Sperm collection and characteristics analysis of the critically endangered Chinese pangolin (*Manis pentadactyla*). Conserv. Physiol. 12:coae010. doi: 10.1093/conphys/coae010, 38957843 PMC11217145

[ref73] Li G. Li W. Song B. Wang C. Shen Q. Li B. . (2021). Differences in the gut microbiome of women with and without hypoactive sexual desire disorder: case control study. J. Med. Internet Res. 23:e25342. doi: 10.2196/25342, 33629964 PMC7952237

[ref74] Li Y. Peng Q. Shang J. Dong W. Wu S. Guo X. . (2023). The role of taurine in male reproduction: physiology, pathology and toxicology. Front. Endocrinol. 14:1017886. doi: 10.3389/fendo.2023.1017886, 36742382 PMC9889556

[ref75] Liang X. Liu J. Di J. Xiao N. Peng Y. Tian Q. . (2024). Toxicity evaluation of processing Evodiae fructus based on intestinal microbiota. Front. Microbiol. 15:1336777. doi: 10.3389/fmicb.2024.1336777, 38435687 PMC10904473

[ref76] Lin P. Ding B. Feng C. Yin S. Zhang T. Qi X. . (2017). *Prevotella* and *Klebsiella* proportions in fecal microbial communities are potential characteristic parameters for patients with major depressive disorder. J. Affect. Disord. 207, 300–304. doi: 10.1016/j.jad.2016.09.051, 27741466

[ref77] Liu C. Hu J. Wu Y. Irwin D. M. Chen W. Zhang Z. . (2021). Comparative study of gut microbiota from captive and confiscated-rescued wild pangolins. J. Genet. Genomics 48, 825–835. doi: 10.1016/j.jgg.2021.07.009, 34474998

[ref78] Lobo A. C. M. Ferreira J. C. P. Kluyber D. Caiaffa M. G. Desbiez A. L. J. De Camilo B. L. . (2025). Pharmacologically assisted semen collection and sperm morphology assessment methods in wild six-banded armadillos (*Euphractus sexcinctus*). Anim. Reprod. Sci. 277:107851. doi: 10.1016/j.anireprosci.2025.107851, 40315592

[ref79] Lundy S. D. Sangwan N. Parekh N. V. Selvam M. K. P. Gupta S. McCaffrey P. . (2021). Functional and taxonomic dysbiosis of the gut, urine, and semen microbiomes in male infertility. Eur. Urol. 79, 826–836. doi: 10.1016/j.eururo.2021.01.014, 33573862

[ref80] Lv M. Wang Y. Qu P. Li S. Yu Z. Qin X. . (2021). A combination of cecum microbiome and metabolome in CUMS depressed rats reveals the antidepressant mechanism of traditional Chinese medicines: a case study of Xiaoyaosan. J. Ethnopharmacol. 276:114167. doi: 10.1016/j.jep.2021.114167, 33984458

[ref81] Ma J. E. Jiang H. Y. Li L. M. Zhang X. J. Li G. Y. Li H. M. . (2018). The Fecal metagenomics of Malayan pangolins identifies an extensive adaptation to myrmecophagy. Front. Microbiol. 9:2793. doi: 10.3389/fmicb.2018.02793, 30532742 PMC6265309

[ref82] Mahmoud M. A. Megahed G. Yousef M. S. Ali F. A. Z. Zaki R. S. Abdelhafeez H. H. (2020). *Salmonella typhimurium* triggered unilateral epididymo-orchitis and splenomegaly in a Holstein bull in Assiut, Egypt: a case report. Pathogens 9:314. doi: 10.3390/pathogens9040314, 32344573 PMC7238186

[ref83] Marchiani S. Baccani I. Tamburrino L. Mattiuz G. Nicolò S. Bonaiuto C. . (2021). Effects of common gram-negative pathogens causing male genitourinary-tract infections on human sperm functions. Sci. Rep. 11:19177. doi: 10.1038/s41598-021-98710-5, 34584150 PMC8478950

[ref84] Martin G. B. Blache D. Miller D. Vercoe P. E. (2010). Interactions between nutrition and reproduction in the management of the mature male ruminant. Anim. 4, 1214–1226. doi: 10.1017/S1751731109991674, 22444618

[ref85] Mason G. J. (2010). Species differences in responses to captivity: stress, welfare and the comparative method. Trends Ecol. Evol. 25, 713–721. doi: 10.1016/j.tree.2010.08.011, 20952089

[ref86] Mayer E. A. Tillisch K. Gupta A. (2015). Gut/brain axis and the microbiota. J. Clin. Invest. 125, 926–938. doi: 10.1172/JCI76304, 25689247 PMC4362231

[ref87] Melis M. R. Sanna F. Argiolas A. (2022). Dopamine, erectile function and male sexual behavior from the past to the present: a review. Brain Sci. 12:826. doi: 10.3390/brainsci12070826, 35884633 PMC9312911

[ref88] Mendoza-Chamizo B. Løbner-Olesen A. Charbon G. (2018). Coping with reactive oxygen species to ensure genome stability in *Escherichia coli*. Genes 9:565. doi: 10.3390/genes9110565, 30469410 PMC6267047

[ref89] Menéndez G. G. Soto J. Barreto J. Gutiérez Á. Soto C. Pérez A. B. . (2023). Randomized clinical trial demonstrate the safety assessment of *Dietzia natronolimnaea* C79793-74 for use as a probiotic in humans. J. Probiotics Health 11:336. doi: 10.20944/preprints202311.1226.v1

[ref90] Mestanza-Ramón C. Henkanaththegedara S. M. Vásconez Duchicela P. Vargas Tierras Y. Sánchez Capa M. Constante Mejía D. . (2020). In-situ and ex-situ biodiversity conservation in Ecuador: a review of policies, actions and challenges. Diversity 12:315. doi: 10.3390/d12080315

[ref91] Meston C. M. Frohlich P. F. (2000). The neurobiology of sexual function. Arch. Gen. Psychiatry 57, 1012–1030. doi: 10.1001/archpsyc.57.11.1012, 11074867

[ref92] Metz A. R. White A. Ripplinger J. Spence Davizon E. Barnes M. (2024). Notes from the field: toxigenic *Corynebacterium ulcerans* in humans and household pets—Utah and Colorado, 2022–2023. MMWR Morb. Mortal Wkly. Rep. 73, 534–535. doi: 10.15585/mmwr.mm7323a3, 38870487 PMC11199016

[ref93] Mir H. D. Milman A. Monnoye M. Douard V. Philippe C. Aubert A. . (2020). The gut microbiota metabolite indole increases emotional responses and adrenal medulla activity in chronically stressed male mice. Psychoneuro 119:104750. doi: 10.1016/j.psyneuen.2020.104750, 32569990

[ref94] Mohapatra R. K. Panda S. (2014). Husbandry, behaviour and conservation breeding of Indian pangolin. Folia Zool. 63, 73–80. doi: 10.25225/FOZO.V63.I2.A4.2014

[ref95] Mohapatra R. Panda S. Nair M. (2015). On the mating behaviour of captive Indian pangolin (*Manis crassicaudata*). Taprobanica 7, 57–59. doi: 10.4038/TAPRO.V7I1.7175

[ref96] Mohapatra R. Panda S. Sahu S. (2018). On the gestation period of Indian pangolins (*Manis crassicaudata*) in captivity. Biodiversity Int. J. 2, 559–560. doi: 10.15406/bij.2018.02.00112

[ref97] Möller J. Busch A. Berens C. Hotzel H. Burkovski A. (2021). Newly isolated animal pathogen Corynebacterium silvaticum is cytotoxic to human epithelial cells. Int. J. Mol. Sci. 22:3549. doi: 10.3390/ijms22073549, 33805570 PMC8037504

[ref98] Morrow G. Nicol S. C. (2009). Cool sex? Hibernation and reproduction overlap in the echidna. PLoS One 4:e6070. doi: 10.1371/journal.pone.0006070, 19562080 PMC2699653

[ref99] Mortimer C. McNeilly A. Fisher R. Murray M. Besser G. (1974). Gonadotrophin-releasing hormone therapy in hypogonadal males with hypothalamic or pituitary dysfunction. Br. Med. J. 4, 617–621. doi: 10.1136/bmj.4.5945.617, 4613417 PMC1612996

[ref100] Mumcu A. Karaer A. Dogan B. Tuncay G. (2020). Metabolomics analysis of seminal plasma in patients with idiopathic Oligoasthenoteratozoospermia using high-resolution NMR spectroscopy. Andrology 8, 450–456. doi: 10.1111/andr.12707, 31520509

[ref101] Oh J. Byrd A. L. Deming C. Conlan S. Kong H. H. Segre J. A. (2014). Biogeography and individuality shape function in the human skin metagenome. Underw. Nat. 514, 59–64. doi: 10.1038/nature13786, 25279917 PMC4185404

[ref102] Okeleji L. O. Ajayi L. O. Odeyemi A. N. Amos V. Akanbi B. G. Onaolapo M. C. . (2024). Bacterial zoonotic diseases and male reproduction. Zoonotic Dis. 4, 97–113. doi: 10.3390/zoonoticdis4010010

[ref103] Olff M. (1999). Stress, depression and immunity: the role of defense and coping styles. Psychiatry Res. 85, 7–15. doi: 10.1016/S0165-1781(98)00139-5, 10195312

[ref104] Olney P. J. S. (2005). Building a Future for Wildlife The World Zoo and Aquarium Conservation Strategy. Bern: WAZA, 7–10.

[ref105] Oyanguren M. Molina E. Mugica M. Ladero-Auñon I. Fuertes M. Fernández M. . (2024). Probiotic bacteria can modulate immune responses to paratuberculosis vaccination. Front. Cell. Infect. Microbiol. 14:1394070. doi: 10.3389/fcimb.2024.1394070, 38895731 PMC11183331

[ref106] Pal R. Athamneh A. I. Deshpande R. Ramirez J. A. Adu K. T. Muthuirulan P. . (2023). Probiotics: insights and new opportunities for Clostridioides difficile intervention. Crit. Rev. Microbiol. 49, 414–434. doi: 10.1080/1040841X.2022.2072705, 35574602 PMC9743071

[ref107] Panjang E. Lim H. Y. Thomas R. J. Goossens B. Hearn A. J. Macdonald D. W. . (2024). Mapping the distribution of the Sunda pangolin (*Manis javanica*) within natural forest in Sabah, Malaysian Borneo. Global Ecol. Conserv. 52:e02962. doi: 10.1016/j.gecco.2024.e02962, 38826717

[ref108] Pearce M. M. Hilt E. E. Rosenfeld A. B. Zilliox M. J. Thomas-White K. Fok C. . (2014). The female urinary microbiome: a comparison of women with and without urgency urinary incontinence. MBio 5, e01283–e01214. doi: 10.1128/mbio.01283-14, 25006228 PMC4161260

[ref109] Perera P. Karawita K. Pabasara M. (2017). Pangolins (*Manis crassicaudata*) in Sri Lanka: a review of current knowledge, threats and research priorities. J. Trop. For. Environ. 7, 1–14. doi: 10.31357/jtfe.v7i1.3018

[ref110] Perez-Carrasco V. Soriano-Lerma A. Soriano M. Gutiérrez-Fernández J. Garcia-Salcedo J. A. (2021). Urinary microbiome: yin and yang of the urinary tract. Front. Cell. Infect. Microbiol. 11:617002. doi: 10.3389/fcimb.2021.617002, 34084752 PMC8167034

[ref111] Podschun R. Ullmann U. (1998). Klebsiella spp. as nosocomial pathogens: epidemiology, taxonomy, typing methods, and pathogenicity factors. Clin. Microbiol. Rev. 11, 589–603. doi: 10.1128/cmr.11.4.589, 9767057 PMC88898

[ref112] Pradhan S. Pradhan R. (2020). How does the Chinese pangolin behave in the wild-observations on activities of Chinese pangolin, *Manis pentadactyla* in the agro ecosystems of Darjeeling, eastern Himalaya, India. ‌Biodivers. Int. J.‌ 4, 175–180. doi: 10.15406/bij.2020.04.00181

[ref113] Prasetiyono B. W. H. E. Ondho Y. S. Subrata A. Pratiwi P. K. Zahra M. B. Itmamulwafa T. . (2020). The effect of choline chloride supplementation on the reproductive performance of simmental bulls fed protected protein in the ration. Buletin Peternakan 44, 83–89. doi: 10.21059/buletinpeternak.v44i2.55338

[ref114] Qin J. Li R. Raes J. Arumugam M. Burgdorf K. S. Manichanh C. . (2010). A human gut microbial gene catalogue established by metagenomic sequencing. Underw. Nat. 464, 59–65. doi: 10.1038/nature08821, 20203603 PMC3779803

[ref115] Qin N. Yang F. Li A. Prifti E. Chen Y. Shao L. . (2014). Alterations of the human gut microbiome in liver cirrhosis. Nature 513, 59–64. doi: 10.1038/nature13568, 25079328

[ref116] Qiu X. Li Z. Huang S. Cai X. Qu S. Zheng Z. . (2023). Electroacupuncture improves depression-like behavior by regulating the abundance of lactobacillus and staphylococci in mice. J. Integr. Neurosci. 22:28. doi: 10.31083/j.jin2202028, 36992578

[ref117] Radford-Smith D. E. Anthony D. C. Benz F. Grist J. T. Lyman M. Miller J. J. . (2023). A multivariate blood metabolite algorithm stably predicts risk and resilience to major depressive disorder in the general population. EBioMedicine 93:104643. doi: 10.1016/j.ebiom.2023.104643, 37327674 PMC10275706

[ref118] Rato L. Alves M. G. Socorro S. Duarte A. I. Cavaco J. E. Oliveira P. F. (2012). Metabolic regulation is important for spermatogenesis. Nat. Rev. Urol. 9, 330–338. doi: 10.1038/nrurol.2012.77, 22549313

[ref119] Reasoner S. A. Francis J. Hadjifrangiskou M. (2025). The urinary microbiome: the next frontier of bacterial ecology. J. Bacteriol. 207:e0010525. doi: 10.1128/jb.00105-25, 40704791 PMC12369349

[ref120] Regan P. C. (1999). Hormonal correlates and causes of sexual desire: a review. Can. J. Hum. Sex. 8, 1–16.

[ref121] Rhim H. C. Kim M. S. Park Y. J. Choi W. S. Park H. K. Kim H. G. . (2019). The potential role of arginine supplements on erectile dysfunction: a systemic review and Meta-analysis. J. Sex. Med. 16, 223–234. doi: 10.1016/j.jsxm.2018.12.002, 30770070

[ref122] Robino L. Navarro N. Canales-Huerta N. González M. J. Cruz E. Sauto R. . (2025). Urogenital microbiome, intracellular bacterial communities, and their contribution to urinary tract infections. Microbiol. Spectrum 13:e0124725. doi: 10.1128/spectrum.01247-25, 41055348 PMC12584659

[ref123] Rondepierre F. Meynier M. Gagniere J. Deneuvy V. Deneuvy A. Roche G. . (2024). Preclinical and clinical evidence of the association of colibactin-producing *Escherichia coli* with anxiety and depression in colon cancer. World J. Gastroenterol. 30, 2817–2826. doi: 10.3748/wjg.v30.i21.2817, 38899326 PMC11185296

[ref124] Rotimi D. E. Iyobhebhe M. Oluwayemi E. T. Olajide O. P. Akinsanola B. A. Evbuomwan I. O. . (2024). Energy metabolism and spermatogenesis. Heliyon 10:e38591. doi: 10.1016/j.heliyon.2024.e38591, 39397940 PMC11470522

[ref125] Scher J. U. Sczesnak A. Longman R. S. Segata N. Ubeda C. Bielski C. . (2013). Expansion of intestinal *Prevotella copri* correlates with enhanced susceptibility to arthritis. eLife 2:e01202. doi: 10.7554/eLife.01202, 24192039 PMC3816614

[ref126] Shahroodian S. Mirshekar M. Talebi M. Torki A. Amirmozafari N. (2022). Association between virulence factors and biofilm formation in *Enterococcus faecalis* isolated from semen of infertile men. Am. J. Reprod. Immunol. 88:e13561. doi: 10.1111/aji.13561, 35499217

[ref127] Shen Z. Xie C. Huang H. Cheng H.-Y. Zheng R. (2024). The captive behavior and reproduction of the Chinese pangolin, *Manis pentadactyla* (Pholidota, Manidae). ARPHA Preprints 5:e142195. doi: 10.3897/arphapreprints.e142195

[ref128] Shen J. Zhao W. Cheng J. Cheng J. Zhao L. Dai C. . (2023). Lipopolysaccharide accelerates tryptophan degradation in the ovary and the derivative kynurenine disturbs hormone biosynthesis and reproductive performance. J. Hazard. Mater. 458:131988. doi: 10.1016/j.jhazmat.2023.131988, 37418963

[ref129] Sheng Z. Liu J. Wang M. Chen X. Xu J. Zhang C. . (2025). Exploring bladder cancer through urinary microbiota: innovative "urinetypes" classification and establishment of a diagnostic model. J. Transl. Med. 23:809. doi: 10.1186/s12967-025-06518-y, 40696353 PMC12285140

[ref130] Shi L. Wang H. Lu Z. (2016). “Staphylococcal infection and infertility,” in Genital Infections and Infertility, (London: Intech), 159–177.

[ref131] Shimada Y. Zang L. Ishimaru T. Nishiura K. Matsuda K. Uchida R. . (2024). Lipid-and glucose-lowering effects of Rhamnan sulphate from Monostroma nitidum with altered gut microbiota in mice. Food Sci. Nutr. 12, 4342–4352. doi: 10.1002/fsn3.4100, 38873438 PMC11167150

[ref132] Shrestha E. White J. R. Yu S. H. Kulac I. Ertunc O. De Marzo A. M. . (2018). Profiling the urinary microbiome in men with positive versus negative biopsies for prostate Cancer. J. Urol. 199, 161–171. doi: 10.1016/j.juro.2017.08.001, 28797714 PMC5937117

[ref133] Shweta F. Gurram P. R. O’Horo J. C. Khalil S. (2021). *Brevibacterium* species: an emerging opportunistic cause of bloodstream infections. Mayo Clin. Proc. 96, 1093–1094. doi: 10.1016/j.mayocp.2021.02.005, 33814079

[ref134] Siddiqui H. Lagesen K. Nederbragt A. J. Jeansson S. L. Jakobsen K. S. (2012). Alterations of microbiota in urine from women with interstitial cystitis. BMC Microbiol. 12:205. doi: 10.1186/1471-2180-12-205, 22974186 PMC3538702

[ref135] Silveira L. F. MacColl G. S. Bouloux P. M. (2002). Hypogonadotropic hypogonadism. Semin. Reprod. Med. 20, 327–338. doi: 10.1055/s-2002-36707, 12536356

[ref136] Smriga M. Kameishi M. Uneyama H. Torii K. (2002). Dietary L-lysine deficiency increases stress-induced anxiety and fecal excretion in rats. J. Nutr. 132, 3744–3746. doi: 10.1093/jn/132.12.3744, 12468617

[ref137] Smriga M. Torii K. (2003). Prolonged treatment with L-lysine and L-arginine reduces stress-induced anxiety in an elevated plus maze. Nutr. Neurosci. 6, 125–128. doi: 10.1080/1028415031000079685, 12722988

[ref138] Socała K. Doboszewska U. Szopa A. Serefko A. Włodarczyk M. Zielińska A. . (2021). The role of microbiota-gut-brain axis in neuropsychiatric and neurological disorders. Pharmacol. Res. 172:105840. doi: 10.1016/j.phrs.2021.105840, 34450312

[ref139] Son H. Baek J. H. Go B. S. Jung D.-H. Sontakke S. B. Chung H. J. . (2018). Glutamine has antidepressive effects through increments of glutamate and glutamine levels and glutamatergic activity in the medial prefrontal cortex. Neuropharmacology 143, 143–152. doi: 10.1016/j.neuropharm.2018.09.040, 30266598

[ref140] Stief C. Benard F. Bosch R. Aboseif S. Nunes L. Lue T. F. . (1989). Acetylcholine as a possible neurotransmitter in penile erection. J. Urol. 141, 1444–1448. doi: 10.1016/S0022-5347(17)41342-5, 2566691

[ref141] Su Y. He L. Hu Z. Li Y. Zhang Y. Fan Z. . (2021). Obesity causes abrupt changes in the testicular microbiota and sperm motility of zebrafish. Front. Immunol. 12:639239. doi: 10.3389/fimmu.2021.639239, 34248933 PMC8268156

[ref142] Sun N. C. Lo F. H. Chan F. T. Lin K. S. Pei K. J. (2024). Inconsistent reproductive cycles and postnatal growth between captive and wild Chinese pangolins and its conservation implications. Global Ecol. Conserv. 54:e03057. doi: 10.1016/j.gecco.2024.e03057, 38826717

[ref143] Sun N. C. Pei K. J. Wu L. Y. (2021). Long term monitoring of the reproductive behavior of wild Chinese pangolin (*Manis pentadactyla*). Sci. Rep. 11:18116. doi: 10.1038/s41598-021-97618-4, 34518626 PMC8438059

[ref144] Tahvilian R. Golesorkhi M. A. Parhoudeh F. Heydarpour F. Hosseini H. Baghshahi H. . (2024). The effect of the combination of ginseng, Tribulus Terrestris, and L-arginine on the sexual performance of men with erectile dysfunction: a randomized, double-blind, parallel, and placebo-controlled clinical trial. J. Pharmacopuncture 27, 82–90. doi: 10.3831/KPI.2024.27.2.82, 38948316 PMC11194517

[ref145] Tamai K. Akashi Y. Yoshimoto Y. Yaguchi Y. Takeuchi Y. Shiigai M. . (2018). First case of a bloodstream infection caused by the genus Brachybacterium. J. Infect. Chemother. 24, 998–1003. doi: 10.1016/j.jiac.2018.06.005, 30007866

[ref146] Tarmizi R. Keng Chee Y. Sipangkui S. Zainuddin Z. Z. Fitri W. N. (2020). The comparison of semen collection in electroejaculation, rectal massage and combination of both methods in the critically endangered Malayan pangolin, *Manis javanica*. Animal (Basel) 10:1948. doi: 10.3390/ani10111948, 33113883 PMC7690726

[ref7001] Tink L. N. Jansen R. Steyn C. (2024). The gross reproductive morphology of the male Temminck’s pangolin Smutsia temminckii (Smuts, 1832). Anat. Histol. Embryol. 53:e13084. doi: 10.1111/ahe.13084, 38944690

[ref147] Tresch M. Watté C. Stengard M. Ritter C. Brodard I. Feyer S. . (2023). Corynebacterium oculi-related bacterium may act as a pathogen and carrier of antimicrobial resistance genes in dogs: a case report. BMC Vet. Res. 19:251. doi: 10.1186/s12917-023-03821-y, 38031130 PMC10763336

[ref148] Van Ee C. (1966). A note on breeding the cape pangolin Manis temniincki at Bloemfontein zoo. Int. Zoo Yearb. 6, 163–164. doi: 10.1111/j.1748-1090.1966.tb01734.x

[ref149] Vasudevan R. (2015). Emergence of UTI causing *Staphylococcus aureus* as a superbug: has the pathogen reduced the options of antimicrobial agents for treatment? EC Microbiol. 1, 88–112.

[ref150] Venyo A. (2020). Salmonella urinary tract infections: a review and update. J. Clin. Nephrol. Res. 7:1099. doi: 10.47739/2379-0652/1099

[ref151] Wallage A. Clarke L. Thomas L. Pyne M. Beard L. Ferguson A. . (2015). Advances in the captive breeding and reproductive biology of the short-beaked echidna (*Tachyglossus aculeatus*). Aust. J. Zool. 63, 181–191. doi: 10.1071/ZO14069

[ref152] Wan S. Yu L. Yang Y. Liu W. Shi D. Cui X. . (2024). Exposure to acrylamide and increased risk of depression mediated by inflammation, oxidative stress, and alkaline phosphatase: evidence from a nationally representative population-based study. J. Affect. Disord. 367, 434–441. doi: 10.1016/j.jad.2024.08.217, 39236889

[ref153] Wang J. Ma D. Luo M. Tan Y. Zhong O. Tian G. . (2022). Effect of spermidine on ameliorating spermatogenic disorders in diabetic mice via regulating glycolysis pathway. Reprod. Biol. Endocrinol. 20:45. doi: 10.1186/s12958-022-00890-w, 35255928 PMC8900360

[ref154] Wang X. Yuan B. Huang H. Zhang X. Liu Y. Hou R. . (2024). Abnormal expression of natural mating behaviour of captive adult giant pandas is related to physiological stress. Conserv. Physiol. 12:coae061. doi: 10.1093/conphys/coae061, 39247179 PMC11377310

[ref155] Wei D. Wu D. Zeng W. Che L. Xu S. Fang Z. . (2022). Arginine promotes testicular development in boars through nitric oxide and putrescine. J. Anim. Physiol. Anim. Nutr. 106, 266–275. doi: 10.1111/jpn.13602, 34212433

[ref156] Wishart D. S. (2019). Metabolomics for investigating physiological and pathophysiological processes. Physiol. Rev. 99, 1819–1875. doi: 10.1152/physrev.00035.2018 31434538

[ref157] Wojick K. B. Langan J. N. Terio K. A. Righton A. Drees R. (2018). Anatomy, histology, and diagnostic imaging of the reproductive tract of male aardvark (*Orycteropus afer*). J. Zoo Wildl. Med. 49, 648–655. doi: 10.1638/2017-0213.1, 30212348

[ref158] Wu Y. Li H. Miao Y. Peng J. Wei H. (2023). Effects of methionine restriction from different sources on sperm quality in aging mice. Nutrients 15:4782. doi: 10.3390/nu15224782, 38004176 PMC10675477

[ref159] Wu G. Ren S. Tang R. Xu C. Zhou J. Lin S.-M. . (2017). Antidepressant effect of taurine in chronic unpredictable mild stress-induced depressive rats. Sci. Rep. 7:4989. doi: 10.1038/s41598-017-05051-3, 28694433 PMC5504064

[ref160] Xiang C. Su L. Han M. Liang J. Hou F. Liao J. (2025). Comparative analysis of gut microbiota and metabolome in captive Chinese and Malayan pangolins. Front. Microbiol. 16:1599588. doi: 10.3389/fmicb.2025.1599588, 40606159 PMC12213675

[ref161] Xie K. Wang C. Scifo E. Pearson B. Ryan D. Henzel K. . (2025). Intermittent fasting boosts sexual behavior by limiting the central availability of tryptophan and serotonin. Cell Metab. 37:1189–1205. e1187. doi: 10.1016/j.cmet.2025.03.001, 40157367

[ref162] Xiong Y. Li B. Wang K. Li J. He S. (2023). Betaine ameliorates heat stress-induced apoptosis by affecting oxidative and endoplasmic reticulum stress in mouse Leydig cells. Biosci. Biotechnol. Biochem. 88, 53–62. doi: 10.1093/bbb/zbad151, 37863837

[ref163] Yan D. Guo X. Zeng X. Jia M. Tao L. Wang X. . (2023). Specific mating behavior of Malayan pangolin (*Manis javanica*) in captivity. Sci. Rep. 13:8592. doi: 10.1038/s41598-023-35391-2, 37237089 PMC10220066

[ref164] Yan D. Zeng X. Jia M. Guo X. Deng S. Tao L. . (2021). Successful captive breeding of a Malayan pangolin population to the third filial generation. Commun. Biol. 4:1212. doi: 10.1038/s42003-021-02760-4, 34675353 PMC8531396

[ref165] Yang J. Lin S. Feng Y. Wu G. Hu J. (2013). Taurine enhances the sexual response and mating ability in aged male rats. Adv. Exp. Med. Biol. 776, 347–355. doi: 10.1007/978-1-4614-6093-0_32, 23392896

[ref166] Yang J. Wu G. Feng Y. Lv Q. Lin S. Hu J. (2010). Effects of taurine on male reproduction in rats of different ages. J. Biomed. Sci. 17, S9–S8. doi: 10.1186/1423-0127-17-S1-S9, 20804629 PMC2994374

[ref167] Yao Y. Qiu X.-J. Wang D.-S. Luo J.-K. Tang T. Li Y.-H. . (2022). Semen microbiota in normal and leukocytospermic males. Asian J. Androl. 24, 398–405. doi: 10.4103/aja202172, 34916474 PMC9295480

[ref168] Yazici K. U. Ozturk Ş. Percinel Yazici K. I Ustundag B. (2024). Altered arginine/agmatine pathway and polyamines in adolescents diagnosed with major depressive disorder. Clin. Psychopharmacol. Neurosci. 22, 624–634. doi: 10.9758/cpn.24.1176, 39420609 PMC11494420

[ref169] Yoo J.-M. Lee B. D. Sok D.-E. Ma J. Y. Kim M. R. (2017). Neuroprotective action of N-acetyl serotonin in oxidative stress-induced apoptosis through the activation of both TrkB/CREB/BDNF pathway and Akt/Nrf2/antioxidant enzyme in neuronal cells. Redox Biol. 11, 592–599. doi: 10.1016/j.redox.2016.12.034, 28110215 PMC5247570

[ref170] Yoo J. J. Shin H. B. Song J. S. Kim M. Yun J. Kim Z. . (2021). Urinary microbiome characteristics in female patients with acute uncomplicated cystitis and recurrent cystitis. J. Clin. Med. 10:1097. doi: 10.3390/jcm10051097, 33807946 PMC7961880

[ref171] Yu C. Li H. Hua L. Che L. Feng B. Fang Z. . (2025). Deciphering the microbiome, lipopolysaccharides, and metabolome interplay: unveiling putrescine’s mechanism for enhancing sperm quality in heat-stressed boar. Theriogenology 236, 60–73. doi: 10.1016/j.theriogenology.2025.01.027, 39919573

[ref172] Yusoff A. M. Tan T. K. Hari R. Koepfli K. P. Wee W. Y. Antunes A. . (2016). De novo sequencing, assembly and analysis of eight different transcriptomes from the Malayan pangolin. Sci. Rep. 6:28199. doi: 10.1038/srep28199, 27618997 PMC5020319

[ref173] Zendri F. Isgren C. M. Sinovich M. Richards-Rios P. Hopkins K. L. Russell K. . (2021). Case report: toxigenic *Corynebacterium ulcerans* diphtheria-like infection in a horse in the United Kingdom. Front. Vet. Sci. 8:650238. doi: 10.3389/fvets.2021.650238, 34141732 PMC8203807

[ref174] Zhang J. Hui Y. Zhou F. Hou J. (2018). Neuroprotective effects of melatonin on erectile dysfunction in streptozotocin-induced diabetic rats. Int. Urol. Nephrol. 50, 1981–1988. doi: 10.1007/s11255-018-1989-4, 30242548

[ref175] Zhang M. Wang X. Ayala J. Liu Y. An J. Wang D. . (2022). Combined urine metabolomics and 16S rDNA sequencing analyses reveals physiological mechanism underlying decline in natural mating behavior of captive giant pandas. Front. Microbiol. 13:906737. doi: 10.3389/fmicb.2022.906737, 36118243 PMC9478395

[ref176] Zhang F. Wu S. Yang L. Zhang L. Sun R. Li S. (2015). Reproductive parameters of the Sunda pangolin, *Manis javanica*. Folia Zool. 64, 129–135. doi: 10.25225/fozo.v64.i2.a6.2015

[ref177] Zhang F. Wu S. Zou C. Wang Q. Li S. Sun R. (2016). A note on captive breeding and reproductive parameters of the Chinese pangolin, *Manis pentadactyla Linnaeus*, 1758. ZooKeys 618, 129–144. doi: 10.3897/zookeys.618.8886, 27853403 PMC5102053

[ref178] Zhang F. Xu N. Wang W. Yu Y. Wu S. (2021). The gut microbiome of the Sunda pangolin (*Manis javanica*) reveals its adaptation to specialized myrmecophagy. PeerJ 9:e11490. doi: 10.7717/peerj.11490, 34141474 PMC8179220

[ref179] Zhang F. Yu J. Wu S. Li S. Zou C. Wang Q. . (2017). Keeping and breeding the rescued Sunda pangolins (*Manis javanica*) in captivity. Zoo Biol. 36, 387–396. doi: 10.1002/zoo.21388, 29148093

[ref180] Zhang F. Yu Y. Yu J. Wu S. Li S. Wang Q. . (2020). Reproductive behavior of the captive Sunda pangolin (*Manis javanica* Desmarest, 1822). Zoo Biol. 39, 65–72. doi: 10.1002/zoo.21526, 31737937

[ref181] Zhao Z. Chen Q. Zhao B. Hannah M. Wang N. Wang Y. . (2020). Relative transmissibility of shigellosis among male and female individuals: a modeling study in Hubei Province, China. Infect. Dis. Poverty 9, 39–16. doi: 10.1186/s40249-020-00654-x, 32299485 PMC7162736

[ref182] Zhao Q. Huang J.-F. Cheng Y. Dai M.-Y. Zhu W.-F. Yang X.-W. . (2021). Polyamine metabolism links gut microbiota and testicular dysfunction. Microbiome 9, 224–218. doi: 10.1186/s40168-021-01157-z, 34758869 PMC8582214

[ref183] Zhao J. Yao Y. Li D. Zhu W. Xiao H. Xie M. . (2023). Metagenome and metabolome insights into the energy compensation and exogenous toxin degradation of gut microbiota in high-altitude rhesus macaques (*Macaca mulatta*). NPJ Biofilms Microbiomes 9:20. doi: 10.1038/s41522-023-00387-3, 37081021 PMC10119431

[ref184] Zhu Y. Wang R. Fan Z. Luo D. Cai G. Li X. . (2023). Taurine alleviates chronic social defeat stress-induced depression by protecting cortical neurons from dendritic spine loss. Cell. Mol. Neurobiol. 43, 827–840. doi: 10.1007/s10571-022-01218-3, 35435537 PMC9958166

