# Supplementary material for: Metagenomics and metabolomics analyses of the mechanism of non-expression of natural mating behavior in captive male Malayan pangolins (Manis javanica)
Source: Front Microbiol. 2026 Jun 11;17:1828282. doi: 10.3389/fmicb.2026.1828282 (PMC13294059; doi:10.3389/fmicb.2026.1828282)
Supplement: Supplementary file 1 [file Table_1.DOCX]

**Brief photos of the captive environment and simplified diagram of the cage and sampling plastic film structure**


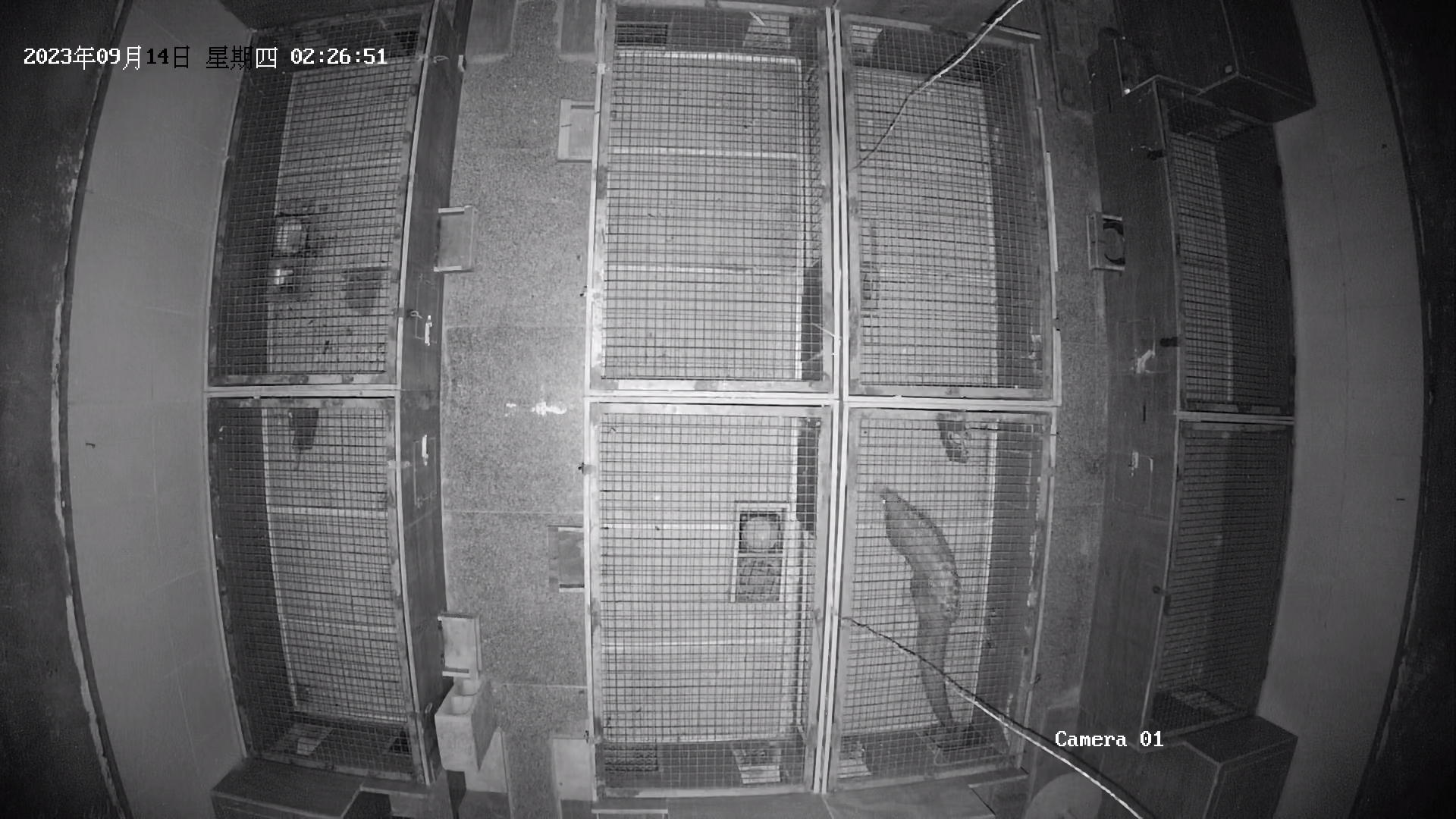


Supplementary figure 1. Brief photos of the captive environment

A

B

C

Supplementary figure 2. Simplified diagram of the cage and sampling plastic film structure.

Note: A. Metal mesh bottom of the cage; B. The slot for the metal mesh bottom of the cage; C. Plastic film.
